# A subchronic history of binge-drinking elicits mild, age- and sex-selective, affective, and cognitive anomalies in C57BL/6J mice

**DOI:** 10.3389/fnbeh.2023.1192076

**Published:** 2023-08-03

**Authors:** C. Leonardo Jimenez Chavez, Eliyana Van Doren, Gavin Scheldrup, Emely Rivera, Jose Torres-Gonzalez, Jessica N. Herbert, Christopher J. E. Denning, Sarah Khorsandi, Andrew Garcia, Marian Castro, Karen K. Szumlinski

**Affiliations:** ^1^Department of Psychological and Brain Sciences, University of California, Santa Barbara, Santa Barbara, CA, United States; ^2^Department of Molecular, Cellular and Developmental Biology, University of California, Santa Barbara, Santa Barbara, CA, United States; ^3^Neuroscience Research Institute, University of California, Santa Barbara, Santa Barbara, CA, United States

**Keywords:** adolescence, Morris water maze, radial arm maze, negative affect, sex differences

## Abstract

**Introduction:**

Alcohol abuse is a risk factor for affective and cognitive disorders, with evidence indicating that adolescent-onset excessive drinking can result in long-term deficiencies in emotional regulation and cognition, with females more susceptible to the negative emotional and cognitive consequences of excessive alcohol consumption. However, our prior examination of the interactions between sex and the age of drinking-onset indicated minimal signs of anxiety-like behavior during alcohol withdrawal, which may have related to the concurrent anxiety testing of male and female subjects.

**Methods:**

The present study addressed this potential confound by assaying for alcohol withdrawal-induced negative affect separately in males and females and expanded our investigation to include measures of spatial and working memory.

**Results:**

Following 14 days of drinking under modified Drinking-in-the-Dark procedures (10, 20, and 40% alcohol v/v; 2 h/day), adolescent and adult binge-drinking mice of both sexes exhibited, respectively, fewer and more signs of negative affect in the light-dark shuttle-box and forced swim tests than their water-drinking counterparts. Adolescent-onset binge-drinking mice also exhibited signs of impaired working memory early during radial arm maze training during early alcohol withdrawal. When tested in late (30 days) withdrawal, only adult female binge-drinking mice buried more marbles than their water-drinking counterparts. However, adolescent-onset binge-drinking mice exhibited poorer spatial memory recall in a Morris water maze.

**Discussion:**

These findings indicate that a subchronic (14-day) binge-drinking history induces mild, age- and sex-selective, changes in negative affect and cognition of potential relevance to understanding individual variability in the etiology and treatment of alcohol abuse and alcohol use disorder.

## Introduction

One of the most common risk factors for the development of dementia and cognitive decline is a history of alcohol abuse (Schwarzinger et al., 2018; Nunes et al., 2019; Wiegmann et al., 2020). Numerous studies have identified that both alcohol use disorder (AUD) and dementia, particularly Alzheimer’s Disease (AD), have a high incidence of cooccurrence (Hersi et al., 2017; Hoffman et al., 2019). Recent evidence suggests that excessive drinking may play a significant role in the development of early-onset dementia and related disorders (Piazza-Gardner et al., 2013; Heymann et al., 2016; Huang et al., 2018; Ledesma et al., 2021). According to evidence from both rodent and human studies, repetitive binge-drinking episodes throughout adolescence are sufficient to generate disruptions within the mesocorticolimbic system that may cause long-term deficiencies in emotional regulation and poor cognitive abilities that become apparent later in adulthood (Novier et al., 2015; Cservenka and Brumback, 2017).

Characterized as stage of rapid neurodevelopment, adolescence normally takes place between 12 and 17 years of age in humans and 28–50 postnatal days (PND) in laboratory mice. Adolescence is commonly recognized as a transitional period marked by the onset of puberty and accompanied by rapid neurobiological, social, and cognitive development (Spear, 2000a,b). As a result of these changes, heightened risk-taking is a hallmark characteristic of adolescence that contributes to the incidence and prevalence of substance use disorders, including AUD (Steinberg, 2007; MacPherson et al., 2010). In contrast to adults, adolescents also exhibit milder affective disturbances and are less vulnerable to the sedative and cognitive deficits that often occur during alcohol withdrawal (Varlinskaya and Spear, 2004; Lee et al., 2016). Thus, research suggests that the perceived advantages of binge-drinking are often more pronounced during this age and such an age-specific attenuation in sensitivity to alcohol’s aversive properties may serve as a permissive factor that contributes to the maintenance of binge drinking patterns among adolescents (Varlinskaya and Spear, 2004; Spear and Varlinskaya, 2005).

The motivational factors that drive drinking to intoxication differ between biological sexes in both humans and laboratory rodents. Evidence suggests that human females are more likely to engage in alcohol binge-drinking to alleviate physical and psychological distress, compared to males (Rodriguez et al., 2020). Although both sexes report a high rate of comorbid mood disorders with AUD, females demonstrate a heighted susceptibility to both the psychological and physiological consequences of excessive drinking (Pollard et al., 2020; Rodriguez et al., 2020). Further, a few findings allude to the notion that females with a history of alcohol abuse experience earlier and greater cognitive-behavioral impairments than their male counterparts (Hebert et al., 2013; Agabio et al., 2017; Ferretti et al., 2018). While several hypotheses attempt to explain why females experience more severe biopsychological effects than males because of alcohol, there is relatively little research that directly examines for sex differences in the effects of excessive drinking on affect or cognitive function, let alone how the age of drinking-onset might interact with biological sex to impact the severity of affective and/or cognitive disturbances during alcohol withdrawal.

Toward this end, we published a study in 2020 designed to examine for sex by age interactions in the expression of negative affect during early (1 day) versus protracted (70 days) alcohol withdrawal in C57BL/6J (B6) mice (Jimenez Chavez et al., 2020). In contrast to other published findings from our laboratory that studied a single sex (e.g., males: Lee et al., 2015, 2016, 2017b, 2018a,b; females: Szumlinski et al., 2019), we detected relatively few behavioral signs of alcohol withdrawal-induced anxiety-like behavior, irrespective of the age of binge-drinking onset. However, when effects of alcohol withdrawal were detected, the magnitude of the effect was comparable between male and female subjects. Two procedural differences might account for the discrepancies in findings between our study of sex differences (Jimenez Chavez et al., 2020) and those employing a single sex (Lee et al., 2016, 2017a,b, 2018a,b; Szumlinski et al., 2019). The first relates to the duration of the alcohol withdrawal period as earlier work compared anxiety-like behavior between 1- and 30-days withdrawal and showed that (at least in adult male B6 mice with a 2-week history of binge-drinking) signs of negative affect dissipate by the 30-day withdrawal time-point (Lee et al., 2017b, 2018a). In contrast, some signs of alcohol-induced negative affect persist for at least 30 days in adult female B6 mice (Szumlinski et al., 2019), but may dissipate at some time between 30 and 70 days withdrawal (Jimenez Chavez et al., 2020). The second procedural difference relates to the concurrent testing of males and females and the potential for sex-related pheromones to influence the affective responses of mice of the opposite sex. Indeed, chemosensory social stimuli, such as those in vaginal secretions, are reported to alter neuronal activity within the mesocorticolimbic system differentially in adolescent versus adult males to affect motivated behavior (Romeo et al., 1998; Bell et al., 2013a,b). Further, exposure to adult female urinary pheromones during testing for anxiety-like behavior produces a testosterone-driven anxiolytic effect in male rats and mice (Aikey et al., 2002; Fernández-Guasti and Martínez-Mota, 2005; Frye et al., 2008). While it is known that affective behavior varies with the estrous cycle in adult female rodents (Fernandez-Guasti and Picazo, 1992), to the best of our knowledge, there is no published report examining how exposure to adult male pheromones might alter anxiety-like behavior in female rodents.

**FIGURE 1:**
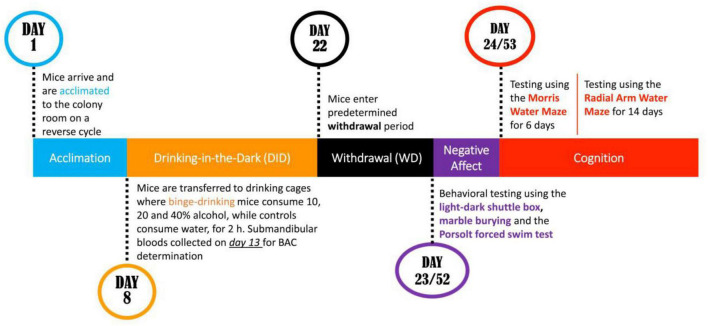
Cartoon of the procedural time-line of the experiments conducted in the present study.

The present study attempted to address both procedural issues by staggering binge-drinking procedures so that anxiety-like behavior was assayed separately in male and female mice on withdrawal days 1 and 30 (respectively, WD1 and WD30). As recent work indicated that mature adult females are more sensitive than their male counterparts to alcohol-induced cognitive impairment (Jimenez Chavez et al., 2022), mice in this study then underwent training under Morris water maze and radial arm water maze procedures to examine for sex by age interactions in alcohol-induced deficits in spatial and working memory in younger adult mice (see Figure 1). Based on the current literature (Szumlinski et al., 2019; Ledesma et al., 2021; Jimenez Chavez et al., 2022), it was hypothesized that alcohol-induced changes in affective and cognitive behavior would be more pronounced in females than males and that a history of binge-drinking during adolescence would induce more robust and/or enduring changes in behavior than that produced by a history of binge-drinking during adulthood.

## Materials and methods

### Subjects

This experiment employed adolescent (postnatal day; PND 21) and adult (PND 49), male and female B6 mice sourced from The Jackson Laboratory (Sacramento, CA, United States). Upon arrival to the vivarium, the mice were immediately housed in groups of four with others of the same age and sex. Mice were allowed 7 days to acclimate to a colony room in a temperature-controlled vivarium under a 12-h reverse light/dark cycle (lights off at 10:00 h). To accommodate space constraints in our vivarium and testing facility, the mice in both withdrawal groups were subdivided into two cohorts, each cohort with a relatively equal number of animals in each group, matched for age, sex and drinking history. In the first cohorts, male mice began drinking a day before the female mice, to ensure that males and females were tested for anxiety-like behavior on different days, thereby minimizing the influence of chemosensory stimuli from the opposite sex; the inverse was done on the subsequent cohorts (Jimenez Chavez et al., 2020, 2022). All animals were identified via tail markings, with access to food and water *ad libitum*, except during the 2-h alcohol-drinking session. In accordance with standard vivarium protocols, drinking cages were lined with sawdust bedding. To minimize any external stressors from unfamiliar handling and changes in the environment, routine cage cleaning activities were halted 5 days before behavioral testing. All experimental methods remained complaint with The Guide for the Care and Use of Laboratory Animals (2014) and all protocols were approved by the Institutional Animal Care and Use Committee of the University of California, Santa Barbara.

### Drinking-in-the-Dark (DID)

A total of 92 mice were subjected to 14-days of binge drinking using a multi-bottle DID procedure that involved concurrent access to unsweetened 10, 20, and 40% (v/v) ethanol. At 13:00 h, all alcohol-drinking mice were relocated from their home cages to individual drinking cages, fitted with a wire lid, located on a free-standing rack in the same colony room within the vivarium. All animals were given 1 h to habituate to their drinking cages prior to alcohol presentation. At 14:00 h, the binge-drinking mice were provided with three alcohol-containing sipper tubes atop the wire cage lid for 2 h (14:00 h–16:00 h), with the position of the sipper tubes randomized each day. As a result of limited space on the freestanding rack, the water-drinking control mice were group housed in drinking cages with their cage mates and received one sipper tube containing water as conducted in comparable studies (e.g., Lee et al., 2018b; Szumlinski et al., 2019; Jimenez Chavez et al., 2020, 2022). Following the 2-h drinking session, all sipper tubes were removed, and the mice were transferred back into their respective home cages. The alcohol-containing sipper tubes were weighed to determine individual consumption. Throughout the drinking period, mice were weighed every 4 days and their weights were utilized to calculate their overall alcohol intake.

### Blood alcohol concentrations

On the 13th day of drinking, submandibular blood samples were collected from the alcohol-drinking mice immediately following their 2-h drinking session. Analytical methods for determining blood alcohol concentrations (BAC) are similar to those employed in our previous studies (Fultz and Szumlinski, 2018; Jimenez Chavez et al., 2020, 2022). Blood samples were stored at −20°C until processing and BACs were determined using headspace gas chromatography. The analysis was performed using a Shimadzu GC-2014 gas chromatography system (Shimadzu, Columbia, MD, USA), and the data was obtained using the GC Solutions 2.10.00 software. To determine the alcohol concentration in each sample, the samples were diluted with non-bacteriostatic saline at a ratio of 1:9, with 50 μl of the sample, and toluene was used as the pre-solvent. The analysis was conducted within 7–10 days of sample collection.

### Behavioral test battery for negative affect

Evidence from our prior work indicated that adolescent male B6 mice with a 2-week history of binge-drinking do not display any noticeable signs of negative affect when tested at 1 day withdrawal (e.g., Lee et al., 2016, 2017b, 2018a,b; Szumlinski et al., 2019; Jimenez Chavez et al., 2020, 2022), we conducted a comprehensive 1-day behavioral test battery including the light-dark shuttle box test, the marble burying test and the Porsolt forced swim test to measure withdrawal-induced changes in negative affect, as detailed below. The order of testing in the various procedures was pseudo-randomized, except for the forced swim test, which occurred last in the test battery in accordance with our animal use protocol. To mitigate any potential impact of chemosensory stimuli from the opposite sex on behavior, we tested males and females on separate days.

#### Light–dark shuttle box test

The light-dark shuttle box test is a behavioral paradigm employed in preclinical research to evaluate anxiety-like behaviors in rodents (Crawley, 1985; Bourin and Hascoët, 2003). In this test, mice are placed in the dark side of a polycarbonate box (46 cm × 22 cm × 24 cm) comprised of two distinct (light vs. dark) environments of equal areas. The light side of the box was white with no lid, while the dark side was black with a black lid. A central divider with an opening allowed the mice to access both sides throughout the 5-min test. The behavioral indices of anxiety-like behaviors, including latency to enter the light side, total time spent in the light side, and the total number entries to the light side, were measured using AnyMaze tracking software (Stoelting Co., Wood Dale, IL, USA). After each testing session, the apparatus was disinfected with Rescue Disinfectant Veterinary Wipes (Virox Animal Health, Oakville, ON, Canada) and the mice were returned to their home cages.

#### Marble burying test

The marble burying test is an established rodent behavioral paradigm that is sensitive to alcohol withdrawal-induced changes in negative affect (Lee et al., 2016, 2017a,b; Szumlinski et al., 2019; Jimenez Chavez et al., 2020, 2022). Mice were placed in a polycarbonate box (12 cm × 8 cm × 6 cm) filled with sterilized sawdust bedding 5 cm deep and 20 round glass black marbles arranged equidistantly in a 4 × 5 square pattern. Animals were allowed to explore the environment and bury marbles for 20 min, where more burying behaviors indicated increased negative affect. After each session, the total number of marbles buried was tallied by the experimenter, the sawdust bedding was replaced with clean bedding, and the mice were returned to their home cages.

#### Porsolt forced swim test

The Porsolt forced swim test is a behavioral paradigm often used to evaluate the reversal of passive coping behavior by antidepressant therapies (Porsolt et al., 2001). The increased swimming behavior observed in this assay can be reversed by pretreatment with anxiolytic agents (Lee et al., 2017a) and therefore, we incorporated it as an additional measure of negative affect. In this assay, mice were placed into a cylindrical glass container (11 cm in diameter) filled with room temperature water for 6 min. Using the AnyMaze tracking software, we measured the latency to the first immobile episode, the total time the animal was immobile, and the number of immobile episodes. Following completion of the test, the mice were returned to their home cages and were monitored until they were dry before returning to the colony room.

### Morris water maze

Following the 1-day test battery for negative affect, conducted on either withdrawal day 1 (WD1) or 30 (WD30), all mice underwent a Morris water maze procedure to assay spatial learning and memory (see Figure 1). The Morris water maze procedures were like those employed previously by our group, using digital video-tracking and AnyMaze software (Lominac et al., 2005; Datko et al., 2017; Jimenez Chavez et al., 2022). The maze is a stainless-steel circular tank (200 cm x 60 cm) containing black intra-maze cues (sun, checkerboard, stripes, moon) one at each four compass coordinate points (N,S,W,E). The tank was filled with room temperature water such that the water level was just above the top of the clear, glass, escape platform. On the first day, a “flag test” was conducted that assayed for visually cued spatial navigation and examined for group differences in swimming speed. For this, a red flag, extending 6 inches above the water, was attached to the escape platform so that the platform location was visible to the mice and the platform was positioned in the NW quadrant. The mice were allowed 2 min to locate the platform and were returned to their home cage upon platform location. If a mouse failed to locate the platform, additional 2-min sessions were conducted until the mouse located the flagged platform. The subsequent 4 days consisted of maze acquisition training, during which the flag was removed and the hidden platform remained situated in the NE quadrant. During acquisition, mice were released from one of the four compass points and allowed 2 min to locate the hidden platform. Once found, mice remained on the platform for 15 s, prior to being returned to the home cage. Once all of the mice completed the first compass point, they were released from the other three compass points so that each mouse underwent four 2-min trials per day. If a mouse failed to locate the hidden platform at any point during maze acquisition, it was guided gently to the platform using long forceps and remained on the platform for 15 sec prior to being returned to the home cage. Twenty-four hours following the fourth acquisition training day, a “probe test” was conducted in which the hidden platform was removed from the tank, and mice swam freely for 2 min and the time spent swimming in the NE quadrant that formerly contained the platform was recorded to index spatial recall. The day following the probe test, a reversal test was conducted in which the hidden platform was positioned in the SW quadrant (i.e., the quadrant opposite to that employed during maze acquisition), and mice underwent four 2-min trials (one for each compass point) in which they were to find the new platform location.

### Water version of the radial arm maze

Following a 1–2 day break, mice were then trained to locate 4 hidden platforms in a water version of the radial arm maze to evaluate working and reference memory. Akin to prior studies (Lominac et al., 2005; Szumlinski et al., 2005; Jimenez Chavez et al., 2022), the maze featured eight arms, four of which had underwater platforms, with the platform locations remaining constant throughout the 14-day training period, but varied for each mouse. Each mouse underwent four, 3-min, trials per day and the trials were conducted in series until the mouse located all four hidden platforms. Upon location of a hidden platform, the mouse remained on the platform for 15 s, at which time it was transferred to a heated holding cage for a 30-s period and the platform was removed from the maze. This was repeated until all four platforms were located. Trained researchers observed the mice throughout each 3-min trial and documented their arm entries in order to calculate the number of reference errors (first entry into an arm that never contained a platform; total of 4 possible), the number of working memory correct errors (entries into an arm that previously contained a platform), the number of working memory incorrect errors (repeated entries into an arm that never contained a platform), chaining behavior (consecutive entries into adjacent arms, irrespective of platform location; a non-spatial navigation strategy) and the time required to locate the platform. The first day of testing was considered a training day and thus was excluded from statistical analysis. The number of each type of error, the number of chains and the time taken during each trial were each summed across the four trials to provide a total for each variable for each training day.

### Replicate study of withdrawal-induced negative affect

The results of the large-scale study described above yielded relatively few signs of alcohol withdrawal-induced negative affect. As assays were conducted concurrently with other testing, we attempted to reduce the influence of any concurrent testing and related personnel traffic in a replicate study more in line with prior studies by our group (e.g., Lee et al., 2016, 2018a,b; Szumlinski et al., 2019). We also single-housed the water-drinking controls during drinking procedures to equate the daily 3-h periods of social isolation across the drinking groups. Otherwise, the drinking and behavioral testing procedures for this replicate study were identical to those employed in the larger scale study described above. Again, males and females were tested for anxiety-like behavior on different days to avoid chemosensory cues from the opposite sex. As the withdrawal-induced negative affect exhibited by adult mice in early withdrawal is robust according to our earlier studies (Lee et al., 2016, 2017a,b; 2018a,b; Szumlinski et al., 2019), we opted to examine behavior at this time-point only in this replicate study with two expectations: (1) adolescent water controls would exhibit more anxiety-like behavior than adults and (2) adult, but not adolescent, alcohol-drinking mice would exhibit signs of anxiety-like behavior. Based on recent work (Jimenez Chavez et al., 2020, 2022), coupled with the majority of results from the present large-scale study (see “Results”), we did not predict any sex difference in the manifestation of withdrawal-induced negative affect. Thus, we employed a sample size of *n* = 6/sex/age/drinking history.

### Statistical analysis

To ensure comparable alcohol intake and BECs between the groups of mice slated to be tested on withdrawal day 1 versus withdrawal day 30 (respectively, WD1 versus WD3), these variables were analyzed using a Sex × Age × Withdrawal ANOVA. The data for alcohol intake in the replicate study was analyzed using a Sex × Age ANOVA. Previous findings from our laboratory suggest that the magnitude of alcohol withdrawal-induced negative affect is influenced by the length of withdrawal (Lee et al., 2016, 2017b, 2018; Szumlinski et al., 2019; Jimenez Chavez et al., 2020). Therefore, to reduce the complexity of the statistical analyses and increase interpretability of the results from the large-scale study, the data for our measures of negative affect and cognitive function were analyzed separately for early (starting on WD1) and late (starting on WD30) withdrawal using a Sex × Age × Drinking History ANOVA. Alpha was set at 0.1 for all analyses as we had *a priori* predictions that: (1) adolescent water-drinking mice would exhibit higher baseline emotionality than their adult counterparts (Lee et al., 2016, 2017a,b); (2) adult binge-drinking mice would exhibit robust signs of negative affect, particularly on WD1 (Lee et al., 2015, 2016, 2017b, 2018a,b; Szumlinski et al., 2019; Jimenez Chavez et al., 2022); and (3) signs of alcohol withdrawal-induced negative affect expressed by adolescent-onset binge-drinkers would be more robust on WD30 compare to WD1 (Lee et al., 2016, 2017b, 2018a,b). For the cognitive data, we conducted Sex x Age x Drinking ANOVAs, with the repeated measures variables of Day/Trial, when appropriate. To increase the statistical power to identify lower-level age and sex differences in our cognitive measures, alpha was set at 0.05 for all analyses and *post hoc* LSD comparisons were performed. For all analyses where sphericity was violated, a Greenhouse–Geisser correction was used. Outliers were identified and excluded from the analyses using the ± 1.5 × IQR rule, however, in instances were too many outliers were identified, we adopted the ± 3 × IQR rule to ensure that only the most extreme outliers were removed. IBM SPSS Statistics software (version 27.0 for Macintosh) was used for all statistical tests, and GraphPad Prism software (version 9.3.1 for Macintosh) was used to create all graphs.

In addition to our primary analyses employing a general linear model, we sought to enhance the comprehensiveness of the data analysis for the large-scale study by employing generalized linear models (GLMs) for our between-subjects analyses. Within this framework, we selected specific GLM types provided by SPSS that were suitable for the nature of our response variables. GLMs are particularly used when assumptions underlying traditional general linear models are violated, allowing for a more flexible modeling approach that adapts to various data distributions and response types (Neal and Simons, 2007; Ng and Cribbie, 2017). For continuous (scale) responses, we implemented two GLM variations: (1) a linear GLM with a normal distribution assumption and the identity link function, and (2) a gamma GLM with a gamma distribution assumption and the logarithmic link function. For count-based response variables, we employed (1) a Poisson loglinear GLM assuming a Poisson distribution and the logarithmic link function, and (2) a negative binomial GLM assuming a negative binomial distribution and the logarithmic link function. Finally, for the dependent variable measuring the number of marbles buried, we utilized a binary logistic GLM with a binomial distribution assumption and the logit link function, as well as a Poisson loglinear GLM. The binary logistic GLM was chosen due to the variable’s bounded maximum value of 20 marbles. Overall, these additional analyses remained consistent with the results from the general linear model (3-way ANOVA; see Tables 1–3).

**TABLE 1:** Comparative analysis of significant statistical results on continuous data for the measures of negative affect and cognition using a general linear model, gamma generalized linear model with log link function (Gamma), and linear generalized linear model (Linear).

Withdrawal day 1						
**Dependent variable**	**General linear model**		**Generalized linear model (Gamma)**		**Generalized linear model (Linear)**	
	**Interaction**	***P*-value**	**Interaction**	***P*-value**	**Interaction**	***P*-value**
Latency to enter the light side	None	all *p*’s > 0.160	None	all *p*’s > 0.169	None	all *p*’s > 0.139
Time spent in the light side	Age × DID Sex × Age Age Effect Sex Effect	0.012 0.040 0.066 0.009	Age × DID Sex × Age Age Effect Sex Effect	0.011 0.022 0.049 0.008	Age × DID Sex × Age Age Effect Sex Effect	0.007 0.029 0.052 0.005
Latency to immobility	DID effect	0.040	DID effect	0.025	DID effect	0.029
Time spent immobile	3-way Inx. Age Effect	0.047 0.019	3-way Inx. Age Effect	0.063 0.027	3-way Inx. Age Effect Sex Effect	0.034 0.012 0.095
Flag test time	None	all *p*’s > 0.500	None	all *p*’s > 0.553	None	all *p*’s > 0.479
Latency to enter platform area	Age × DID	0.021	Age × DID Sex × Age	0.007 0.056	Age × DID	0.014
Time in the probe test	None	all *p*’s > 0.221	None	all *p*’s > 0.215	None	all *p*’s > 0.198
**Withdrawal day 30**						
Latency to enter the light side	None	all *p*’s > 0.228	None	all *p*’s > 0.247	None	all *p*’s > 0.204
Time spent in the light side	None	all *p*’s > 0.140	None	all *p*’s > 0.162	None	all *p*’s > 0.119
Latency to immobility	Sex Effect	0.006	Sex Effect	0.004	Sex × DID Sex Effect	0.084 0.003
Time spent immobile	Sex × DID Age Effect Sex Effect	0.072 0.032 0.003	Sex × DID Age Effect Sex Effect	0.059 0.033 0.003	Sex × DID Age Effect Sex Effect	0.057 0.022 0.002
Flag test time	None	all *p*’s > 0.222	None	all *p*’s > 0.222	None	all *p*’s > 0.199
Latency to enter platform area	None	all *p*’s > 0.461	None	all *p*’s > 0.441	None	all *p*’s > 0.281

**TABLE 2:** Comparative analysis of significant statistical results on count data for the measures of negative affect and cognition using a general linear model, poisson generalized linear model with log as the link function (Poission loglinear), and negative binomial generalized linear model with log as the link function (Negative binomial).

Withdrawal day 1						
**Dependent variable**	**General linear model**		**Generalized linear model (Poisson loglinear)**		**Generalized Linear model (Negative binomial)**	
	**Interaction**	***P*-value**	**Interaction**	***P*-value**	**Interaction**	***P*-value**
Entries to the light side	Age × DID Sex × DID	0.032 0.062	Age × DID Sex × DID	0.033 0.065	None	all *p*’s > 0.621
Immobile episodes	3-way Inx. Age Effect	0.005 0.034	3-way Inx. Age Effect Sex Effect	<0.001 <0.001 0.036	None	all *p*’s > 0.256
Entries to platform area	None	all *p*’s > 0.386	None	all *p*’s > 0.286	None	all *p*’s > 0.710
**Withdrawal day 30**						
Entries to the light side	Sex Effect	0.003	3-way Inx. Age x DID Sex Effect	0.086 0.098 <0.001	None	all *p*’s > 0.352
Immobile episodes	Age × DID Sex × DID Sex Effect	0.094 0.052 0.007	Age × DID Sex × DID DID Effect Sex Effect	0.044 0.011 0.076 <0.001	None	all *p*’s > 0.402
Entries to platform area	Age × DID	0.012	Age × DID	0.001	None	all *p*’s > 0.280

**TABLE 3:** Comparative analysis of significant statistical results on count data for the number of marbles buried in the marble burying test using a general linear model, binary logistic generalized linear model with logit as the link function (Binary logistic), and poisson generalized linear model with log as the link function (Poisson loglinear).

Withdrawal day 1						
**Dependent variable**	**General Linear model**		**Generalized Linear model (Binary logistic)**		**Generalized Linear model (Poisson loglinear)**	
	**Interaction**	***P*-value**	**Interaction**	***P*-value**	**Interaction**	***P*-value**
Number of marbles buried	Age Effect	0.048	Age Effect Sex Effect	<0.001 0.045	Age Effect	0.003
**Withdrawal day 30**						
Number of marbles buried	Age Effect Sex Effect	0.055 0.002	3-way Inx. Sex × DID DID Effect Age Effect Sex Effect	0.002 0.025 0.041 <0.001 <0.001	3-way Inx. Sex × DID DID Effect Age Effect Sex Effect	0.013 0.068 0.072 0.006 < 0.001

To address concerns pertaining to sphericity and homogeneity of variance, we re-analyzed our mixed-model ANOVA results using multilevel models. In contrast with traditional mixed-model ANOVAs, multilevel models do not make assumptions of sphericity or homogeneity of variance (Quené and Van den Bergh, 2004). Moreover, multilevel models are more robust than traditional ANOVAs to violations of distributional assumptions (Schielzeth et al., 2020). These analyses employed a random intercept model, with observations nested within subjects. For ease of interpretation, the Day/Trial variable was treated as a continuous parameter. Overall, the pattern of results resembled those found using traditional mixed model ANOVAs, with only minor exceptions (see Table 4). These statistical analyses were performed in R, utilizing the lmerTest and lme4 packages. As the results of the multilevel model approach failed to yield results that were much different from the mixed-model ANOVA, the data for the replicate study were analyzed using a mixed-model ANOVA, adjusting for violations of sphericity and homogeneity of variance.

**TABLE 4:** Comparative analysis of significant statistical results on continuous cognitive data using a general linear mixed model (Mixed model) versus a multilevel model nested within subjects.

Withdrawal day 1				
**Dependent variable**	**General linear model (Mixed model)**		**Multilevel model (Nested within subjects)**	
	**Interaction**	***P*-value**	**Interaction**	***P*-value**
Acquisition time in the Morris water maze	Day Effect	<0.001	Day Effect	<0.001
Time in the reversal test	Day Effect	<0.001	Day Effect	0.005
Number of reference memory errors	Day × Age Day Effect	0.020 0.004	None	0.124
Number of working memory correct errors (WMC)	4-way Inx. Day Effect	0.041 <0.001	4-way Inx. Day × Sex × DID	0.012 0.029
Number of Working Memory Incorrect Errors (WMI)	Day × Age × DID Day × DID Day Effect	0.025 0.037 <0.001	Day × Age × DID Day Effect	0.012 0.004
Number of chaining episodes	Day × Sex × DID Day × Sex Day Effect	0.016 0.005 <0.001	None	0.102
Time in the Radial Arm Maze	Day Effect	<0.001	4-way Inx. Day × Age × DID Day Effect	0.029 0.045 0.011
**Withdrawal day 30**				
Acquisition time in the Morris water maze	Day × Age Day Effect	0.009 <0.001	Day × Age Age Effect Day Effect	0.006 <0.001 0.046
Time in the reversal test	Day × Age Day Effect	0.005 <0.001	Day × Age Age Effect	0.002 0.003
Number of reference memory errors	Day × Age Day Effect	0.020 0.004	None	0.124
Number of working memory correct errors (WMC)	Day × DID Day Effect	<0.001 <0.001	4-way Inx. Day × Sex × DID	0.017 0.021
Number of working memory incorrect errors (WMI)	Day × Age × DID Day × DID Day Effect	0.025 0.037 <0.001	Day × Age × DID Day Effect	0.012 0.004
Number of chaining episodes	Day × Sex Day Effect	0.036 <0.001	None	0.129
Time in the radial arm maze	Day × DID Day Effect	0.001 <0.001	4-way	0.046

## Results

### Alcohol intake and BECs

**FIGURE 2:**
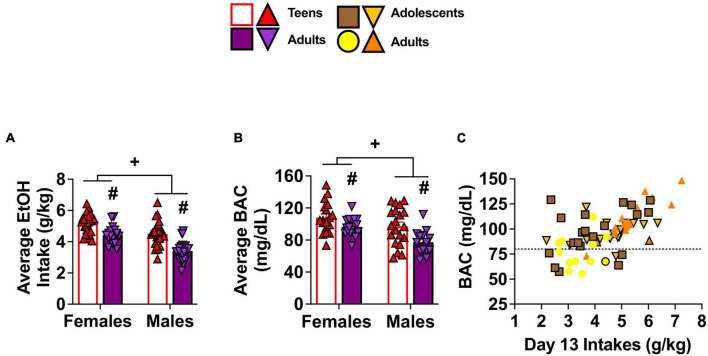
Depiction of age and sex differences in alcohol intake and corresponding BACs. As there were no Withdrawal effects or interactions, the data for alcohol intake and BACs are collapsed across mice slated to be tested on WD1 and WD30. **(A)** On average, adolescent (Adol.) mice consumed more alcohol than adult mice and females consumed more than males [females: adolescents (*n* = 24), adults (*n* = 20); males: adolescents (*n* = 24), adults (*n* = 24)]. **(B)** The average BAC levels obtained on Day 13 of drinking paralleled group differences in alcohol intake [females: adolescents (*n* = 18), adults (*n* = 18); males: adolescents (*n* = 20), adults (*n* = 14)] and **(C)** a positive correlation was observed between BACs and alcohol consumption on Day 13 of drinking [sample sizes same as panel **(B)**]. The data in panels **(A,B)** are presented as the means ± SEMs for the respective number of mice indicated above. ^+^*p* < 0.05, Female vs. Male (main Sex effect); ^#^*p* < 0.05, adolescents vs. adults (main Age effect).

A univariate Sex × Age × Withdrawal ANOVA was conducted to determine group differences in the amount of alcohol consumed during the 14 days of drinking and to confirm equivalent intakes between mice slated to be tested for behavior on WD1 and WD30. While a statistically significant main effect of Withdrawal was observed [*F*(1,84) = 3.99, *p* = 0.049, η^2^p = 0.045], its practical significance may be limited due to the relatively weak effect size and the unequal sample sizes in our study. As such, the data are presented as collapsed across the two withdrawal time-points in Figure 2. Adolescent mice exhibited higher alcohol intake than adult mice [Figure 2A; Age effect *F*(1,84) = 45.491, *p* < 0.001, η^2^p = 0.351], as well as higher alcohol intake by female mice than males [Figure 2A; Sex effect *F*(1,84) = 40.326, *p* < 0.001, η^2^p = 0.324]. No significant 3-way interaction was observed for the average alcohol intake (*p* = 0.754, η^2^p = 0.001) and no other significant interactions were observed (all *p’s* > 0.066).

The average BEC attained on Day 13 of drinking (Figure 2B) exhibited a pattern of group differences that was comparable to that of the average alcohol intake of the mice [Age effect: *F*(1,62) = 15.05, *p* < 0.001, η^2^p = 0.195; Sex effect: *F*(1,62) = 10.06, *p* = 0.002, η^2^p = 0.140] and consistent with this, a Pearson’s correlation showed a positive relationship between BEC levels and alcohol intake (*r* = 0.59, p < 0.001, Figure 2C).

### Light dark box shuttle test

#### Latency to first enter light side

**FIGURE 3:**
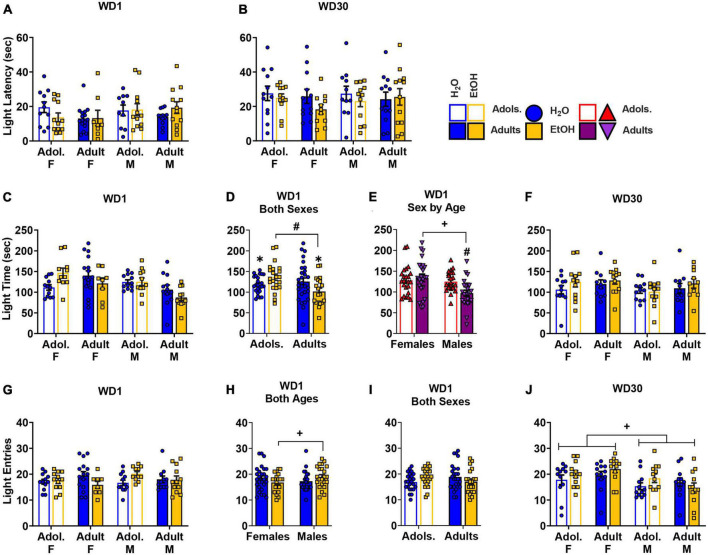
Depiction of the results of the Sex × Age × Drinking History ANOVAs conducted for behavior in the light dark box shuttle test. No group differences were observed for the latency to enter the light side of the shuttle box on either WD1 **(A)** [females: adolescents/Adol. H2O (*n* = 11), EtOH (*n* = 12); adults H2O (*n* = 16), EtOH (*n* = 8); males: adolescent H2O (*n* = 10), EtOH (*n* = 12); adults H2O (*n* = 12), EtOH (*n* = 12)] or WD30 **(B)** [females: adolescents H2O (*n* = 12), EtOH (*n* = 12); adults H2O (*n* = 12), EtOH (*n* = 11); males: adolescent H2O (*n* = 11), EtOH (*n* = 12); adults H2O (*n* = 12), EtOH (*n* = 12)]. **(C)** Summary of the results for the time spent in the light side for all groups tested on WD1 [females: adolescents H2O (*n* = 12), EtOH (*n* = 11); adults H2O (*n* = 16), EtOH (*n* = 8); males: adolescent H2O (*n* = 12), EtOH (*n* = 11); adults H2O (*n* = 12), EtOH (*n* = 11)]. **(D)** On WD1, an Age by Drinking History interaction was observed for the time spent in the light side that reflected less time spent by adult binge-drinking (EtOH) mice versus both adult water (H2O) and adolescent (Adol.) EtOH mice. Additionally, adolescent H2O mice spent less time in the light side than their age- matched EtOH counterparts [adolescents: H2O (*n* = 24), EtOH (*n* = 22); adults: H2O (*n* = 28), EtOH (*n* = 19)]. **(E)** Also on WD1, we detected a Sex by Age interaction that reflected more time spent on the light side by adult females (F) versus adult males (M), while no sex difference was apparent in adolescent mice. Adolescent males, however, spent more time in the light side compared to the adult males [females: adolescents (*n* = 23), adults (*n* = 24); males: adolescents (*n* = 23), adults (*n* = 23)]. **(F)** On WD30, no group differences were detected for the total time spent in the light side of the shuttle box [females: adolescents H2O (*n* = 12), EtOH (*n* = 12); adults H2O (*n* = 11), EtOH (*n* = 12); males: adolescent H2O (*n* = 12), EtOH (*n* = 12); adults H2O (*n* = 11), EtOH (*n* = 10). **(G)** Results for the total number of entries into the light side of the shuttle box test indicated significant interactions on WD1 between Sex by Drinking History and Age by Drinking History [females: adolescents H2O (*n* = 12), EtOH (*n* = 12); adults H2O (*n* = 15), EtOH (*n* = 8); males: adolescent H2O (*n* = 12), EtOH (*n* = 11); adults H2O (*n* = 11), EtOH (*n* = 12)]. **(H)** Follow-up analysis of the Sex by Drinking History interaction revealed that male EtOH mice exhibited more entries into the light side compared to female EtOH mice [females: H2O (*n* = 27), EtOH (*n* = 20); males: H2O (*n* = 23), EtOH (*n* = 23)]. **(I)** The Age by Drinking History interaction on WD1 did not reflect any significant effect of EtOH in either age group [adolescents: H2O (*n* = 24), EtOH (*n* = 23); adults: H2O (*n* = 26), EtOH (*n* = 20)]. **(J)** On WD30, female mice exhibited a greater number of entries into the light side compared to male mice, irrespective of age or drinking condition [females: adolescents H2O (*n* = 1)2, EtOH (*n* = 12); adults H2O (*n* = 12), EtOH (*n* = 12); males: adolescent H2O (*n* = 12), EtOH (*n* = 12); adults H2O (*n* = 12), EtOH (*n* = 12). The data represent the means ± SEMs for the number of mice indicated above. ^+^*p* < 0.10, Female vs. Male; ^#^*p* < 0.10, adolescents vs. adults.

A Sex × Age × Drinking History ANOVA failed to detect any significant differences for the latency to first enter the light-side of the light-dark shuttle-box on either WD1 (Figure 3A) (3-way ANOVA: *p* = 0.883, η^2^*p* = 0.000; all other *p’s* > 0.160) or WD30 (Figure 3B; 3-way ANOVA: *p* = 0.330, η^2^*p* = 0.011, all other *p’s* > 0.228).

#### Time in the light side

On WD1, an Age × Drinking History interaction [*F*(1,85) = 6.65, *p* = 0.012, η^2^p = 0.073] and a Sex × Age interaction [*F*(1,85) = 4.35, *p* = 0.040, η^2^p = 0.049] were found for the time spent in the light side (Figure 3C). As illustrated in Figure 3D, the Age × Drinking History interaction reflected less time spent in the light-side by adult binge-drinking mice versus both adult water controls (*p* = 0.069, *d* = 0.554) and adolescent binge-drinking mice (*p* = 0.004, *d* = 0.935). Adolescent water control mice also spent less time in the light side when compared to their binge-drinking counterparts (*p* = 0.075, *d* = 0.532). The Sex × Age interaction (Figure 3E) reflected more time spent in the light-side by adult female versus adult male mice (*p* = 0.001, *d* = 1.001), with no sex difference apparent in adolescent animals (*p* = 0.680, *d* = 0.122). Additionally, adolescent males spent more time in the light-side compared to the adult males (*p* = 0.006, *d* = 0.832). On WD30, no significant effects or interactions were detected (Figure 3F; 3-way ANOVA: *p* = 0.396, η^2^*p* = 0.009; all other *p’s* > 0.140).

#### Light side entries

On WD1, a Sex × Age × Drinking History ANOVA detected a significant Sex × Drinking History [*F*(1,85) = 3.59, *p* = 0.062, η^2^p = 0.041] and an Age × Drinking History interaction [*F*(1,85) = 4.75, *p* = 0.032, η^2^p = 0.053] for the number of entries into the light-side (Figure 3G). As illustrated in Figure 3H, the Sex × Drinking History interaction reflected a higher number of light side entries in male binge-drinking mice versus the female binge-drinking mice (*p* = 0.072, *d* = 0.563). Although inspection of Figure 3I suggested that adolescent binge-drinking mice made more light side entries than their water controls, while the opposite was true for adult binge-drinking mice, deconstruction of the Age × Drinking History interaction indicated no significant Water-EtOH difference in the adolescent or adult mice (Adolescents: *p* = 0.119, *d* = 0.459; Adults: *p* = 0.135, *d* = 0.456). On WD30, a main Sex effect was observed for the number of light-side entries [*F*(1,88) = 9.48, *p* = 0.003, η^2^p = 0.097; all other *p’s* > 0.203], with females entering the light-side more, overall, than males (Figure 3J).

#### Marble burying test

**FIGURE 4:**
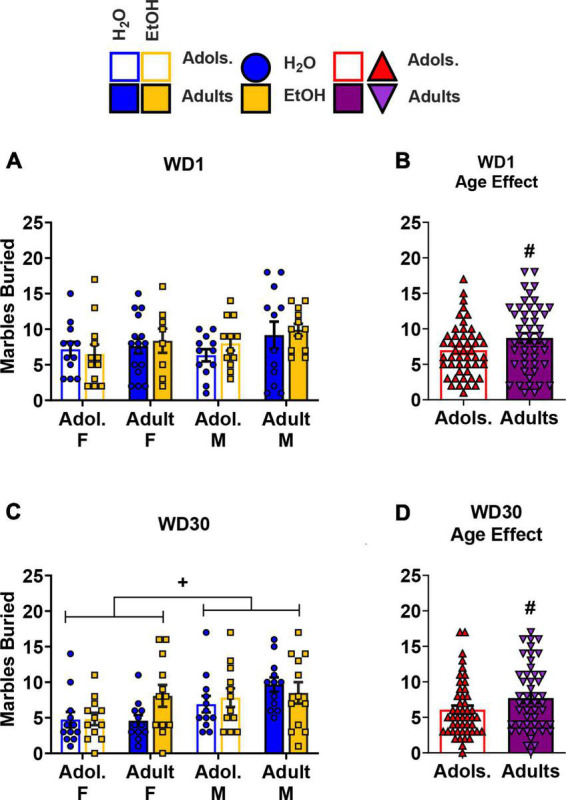
Depiction of the results of the Sex × Age × Drinking History ANOVAs conducted for behavior in the marble burying test. **(A)** On WD1, no significant interactions were observed for the number of marbles buried [females: adolescents/Adol. H2O (*n* = 12), EtOH (*n* = 12); adults H2O (*n* = 16), EtOH (*n* = 8); males: adolescent H2O (*n* = 12), EtOH (*n* = 12); adults H2O (*n* = 12), EtOH (*n* = 12)]. However, **(B)** adult mice buried more marbles than the adolescent mice [adolescents (*n* = 48), adults (*n* = 48)]. **(C)** On WD30, females buried a greater number of marbles compared to the male mice [females: adolescents H2O (*n* = 12), EtOH (*n* = 12); adults H2O (*n* = 12), EtOH (*n* = 12); males: adolescent H2O (*n* = 12), EtOH (*n* = 12); adults H2O (*n* = 12), EtOH (*n* = 12)]. **(D)** Similar to the mice in WD1, adult mice buried more marbles than their adolescent counterparts [adolescents (*n* = 48), adults (*n* = 48)]. The data represent the means ± SEMs for the number of mice indicated above. ^+^*p* < 0.10, Female vs. Male; ^#^*p* < 0.10, adolescents vs. adults.

The data for the number of marbles buried on WD1 by all of the groups are presented in Figure 4A. An analysis of these data indicated more marbles buried by adult versus adolescent mice (Figure 4B) [Age effect: *F*(1,88) = 4.01, *p* = 0.048, η^2^p = 0.044], but no other effects or interactions were found at this withdrawal time-point (Sex × Age × Drinking History ANOVA: *p* = 0.511, η^2^p = 0.005; all other *p’s* > 0.496). The data for the number of marbles buried on WD30 by all of the groups are presented in Figure 4C. For these mice, no significant interactions were found [3-way ANOVA, *p* = 0.104, η^2^p = 0.030; all other interactions *p*’s > 0.255]. However, significant main effects of Sex (Figure 4C) and Age (Figure 4D) were detected [Sex effect: *F*(1,88) = 10.16, *p* = 0.002, η^2^p = 0.104]; Age effect: *F*(1,88) = 3.77, *p* = 0.055, η^2^p = 0.41], indicating that females buried more marbles versus the male mice, and adult mice buried more marbles compared to their adolescent counterparts.

### Porsolt forced swim test

#### Latency to first immobile episode

**FIGURE 5:**
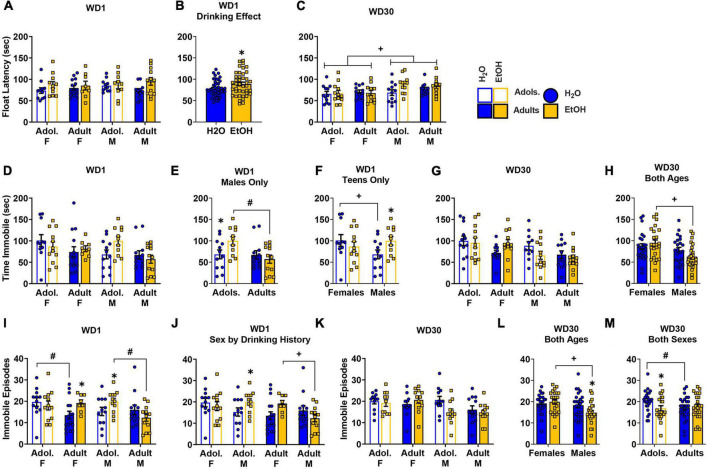
Depiction of the results of the Sex × Age × Drinking History ANOVAs conducted for behavior in the Porsolt forced swim test. **(A)** On WD1, we detected no significant 3-way interaction for the latency to immobility [females: adolescents/Adol. H2O (*n* = 11), EtOH (*n* = 11); adults H2O (*n* = 14), EtOH (*n* = 8); males: adolescent H2O (*n* = 11), EtOH (*n* = 10); adults H2O (*n* = 11), EtOH (*n* = 12)]. **(B)** However, binge-drinking (EtOH) mice had a longer latency to immobility, overall, than water (H2O) mice, on WD1 [sample size. **(C)** Overall, males (M) exhibited a longer latency to immobility on WD30 than females (F) [females: adolescents H2O (*n* = 12), EtOH (*n* = 12); adults H2O (*n* = 11), EtOH (*n* = 11); males: adolescent H2O (*n* = 12), EtOH (*n* = 12); adults H2O (*n* = 12), EtOH (*n* = 10)]. **(D)** On WD1, a significant 3-way interaction was detected for the time spent immobile, that reflected a longer time spent immobile by adolescent (Adol.) EtOH males versus both adolescent H2O and adult EtOH males **(E)** [females: adolescents H2O (*n* = 11), EtOH (*n* = 12); adults H2O (*n* = 14), EtOH (*n* = 8); males: adolescent H2O (*n* = 12), EtOH (*n* = 11); adults H2O (*n* = 12), EtOH (*n* = 12)]. **(F)** When deconstructed along the Age factor, adolescent male EtOH mice spent more time immobile than their H2O counterparts [females: H2O (*n* = 11), EtOH (*n* = 12); males: H2O (*n* = 12), EtOH (*n* = 11). **(G)** For WD30, no significant 3-way interaction was detected for the time spent immobile [females: adolescents H2O (*n* = 12), EtOH (*n* = 12); adults H2O (*n* = 12), EtOH (*n* = 12); males: adolescent H2O (*n* = 11), EtOH (*n* = 12); adults H2O (*n* = 12), EtOH (*n* = 12)]. **(H)** However, a Sex by Drinking History interaction found that female EtOH mice spent more time immobile than male EtOH mice [females: H2O (*n* = 24), EtOH (*n* = 24); males: H2O (*n* = 23), EtOH (*n* = 24)]. **(I)** A significant Sex by Age by Drinking History interaction was observed for the number of immobile episodes on WD1, and results deconstructed along the Sex factor revealed that adolescent male EtOH mice had more immobile episodes than their H2O counterparts and adult EtOH males, while adult female EtOH mice also had more episodes than their H2O counterparts [females: adolescents H2O (*n* = 12), EtOH (*n* = 12); adults H2O (*n* = 16), EtOH (*n* = 8); males: adolescent H2O (*n* = 12), EtOH (*n* = 12); adults H2O (*n* = 12), EtOH (*n* = 12)]. **(J)** Analysis along the Age factor identified sex-related differences where adult female EtOH had more immobile episodes than adult male EtOH, and adolescent male EtOH had more immobile episodes than their H2O counterparts [sample sizes same as panel **(J)**]. **(K)** For WD30, a significant Sex by Drinking History and Age by Drinking History interaction were detected [females: adolescents H2O (*n* = 12), EtOH (*n* = 11); adults H2O (*n* = 12), EtOH (*n* = 12); males: adolescent H2O (*n* = 12), EtOH (*n* = 12); adults H2O (*n* = 12), EtOH (*n* = 12)]. **(L)** Follow-up analyses revealed that male H2O mice had more immobile episodes than their EtOH counterparts and that females EtOH mice had more immobile episodes than their EtOH male counterparts [females: H2O (*n* = 24), EtOH (*n* = 23); males: H2O (*n* = 24), EtOH (*n* = 24)]. **(M)** An Age by Drinking History interaction indicated that adolescent H2O mice had more immobile episodes than their EtOH counterparts and the adult H2O mice [adolescents: H2O (*n* = 24), EtOH (*n* = 23); adults: H2O (*n* = 24), EtOH (*n* = 24)]. The data represent the means ± SEMs for the number of mice indicated above. *p < 0.10, EtOH vs. H2O; ^+^*p* < 0.10, Female vs. Male; ^#^*p* < 0.10, adolescents vs. adults.

The data for the latency to first float in the forced swim test on WD1 are presented in Figure 5A. A Sex × Age × Drinking History ANOVA detected no interactions with respect to the latency to first float in the forced swim test on WD 1 [Sex × Age Drinking History ANOVA: *p* = 0.161, η^2^p = 0.024, all other interactions *p*’s > 0.525]. However, a significant main effect of Drinking History was detected (Figure 5B) [*F*(1,80) = 4.34, *p* = 0.040, η^2^p = 0.051] that reflected a longer latency to immobility in binge-drinking mice, relative to their water-drinking counterparts. For the mice tested on WD30, a significant main effect of Sex [*F*(1,84) = 8.07, *p* = 0.006, η^2^p = 0.088] reflected a shorter immobile latency for females versus males, irrespective of their binge-drinking history or age of binge-drinking onset (Figure 5C; all other *p’s* > 0.102).

#### Time spent immobile

The data for the time spent immobile during the forced swim test on WD1 are presented in Figure 5D. On WD1, a significant Sex x Age x Drinking History interaction was observed for the total time spent immobile during the forced swim test [*F*(1,84) = 4.08, *p* = 0.047, η^2^p = 0.046]. To investigate potential age differences, this interaction was split along the Sex factor and revealed a significant Age x Drinking History interaction for the male mice (Figure 5D, right) [*F*(1,43) = 4.41, *p* = 0.042, η^2^p = 0.093], but no significant main effect or interactions for the females (Figure 5D, left) [ANOVA: *p* = 0.378, η^2^p = 0.019]. As illustrated in Figure 5E, adolescent male binge-drinking mice spent more time immobile than their water-drinking counterparts (*p* = 0.031, *d* = 0.032) and the adult male binge-drinking mice (*p* = 0.004, *d* = 1.260). To analyze for sex-related differences in the time spent immobile, the 3-way interaction was also deconstructed along the Age variable. This deconstruction found a Sex × Drinking History interaction for the adolescent mice, but not for the adult mice [Adolescent: *F*(1,42) = 4.08, *p* = 0.050, η^2^p = 0.089; Adult ANOVA: *p* = 0.419, η^2^p = 0.016]. As illustrated in Figure 5F (left vs. right), adolescent female water-drinking mice spent more time immobile than their male counterparts (*p* = 0.050, *d* = 0.844). Additionally, the adolescent male binge-drinking mice also spent more time immobile than the water-drinking control mice (Figure 5F, right; *p* = 0.055, *d* = 0.823).

The data for the time spent immobile on WD30 is presented in Figure 5G. On WD30, a Sex × Drinking History interaction was found for the total time spent immobile [*F*(1,87) = 3.33, *p* = 0.072, η^2^p = 0.037]. This interaction reflected a longer time spent immobile by female binge-drinking mice compared to the male binge-drinking mice (Figure 5H; *p* = 0.001, *d* = 0.991). No other significant interactions were observed for this variable on WD30 [3-way ANOVA: *p* = 0.641, η^2^p = 0.003; all other *p’s* > 0.132).

#### Immobile episodes

The data for the number of immobile episodes on WD1 are presented in Figure 5I. A 3-way Sex × Age × Drinking History interaction was revealed for this variable [*F*(1,88) = 8.29, *p* = 0.005, η^2^p = 0.086]. To examine for age differences, the interaction was first deconstructed along the Sex factor, which resulted in significant Age × Drinking History interactions for both male [*F*(1,44) = 5.05, *p* = 0.030, η^2^p = 0.103] and female subjects [*F*(1,44) = 3.39, *p* = 0.072, η^2^p = 0.072]. As illustrated for males in Figure 5I (right), the 2-way interaction reflected a higher number of immobile episodes for the adolescent binge-drinking mice versus their water-drinking counterparts (*p* = 0.082, *d* = 0.727). Additionally, adolescent male binge-drinking mice had a higher number of immobile episodes versus adult binge-drinking males (*p* = 0.006, *d* = 1.81). In contrast, as illustrated in Figure 5I (left), the 2-way interaction detected in females reflected water-alcohol differences for adult mice only (*p* = 0.056, *d* = 0.849). We also observed a higher number of immobile episodes for adolescent water-drinking females versus their adult counterparts (*p* = 0.021, *d* = 0.911). To examine for sex-related differences in basal and withdrawal-induced behavior, the 3-way interaction was analyzed also along the Age factor. This deconstruction revealed a significant Sex × Drinking History interaction for both adult [*F*(1,44) = 5.603, *p* = 0.022, η^2^p = 0.113] and adolescent mice [*F*(1,44) = 2.841, *p* = 0.099, η^2^p = 0.061]. Thus, the data in Figure 5I was re-arranged to better illustrate the age-dependency of these sex differences (Figure 5J). As illustrated in Figure 5J (right), the Sex × Drinking History interaction in adult mice reflected a sex difference in binge-drinkers, but not water controls, where adult female binge-drinkers had more immobile episodes versus the adult male binge-drinking mice (*p* = 0.028, *d* = 1.035). For the adolescent mice (Figure 5J, left), no significant water-alcohol differences were observed in female mice, however, adolescent male binge-drinking mice had more immobile episodes than their water-drinking counterparts (*p* = 0.086, *d* = 0.717).

The data for the number of immobile episodes on WD30 are presented in Figure 5K. On WD30, significant Sex × Drinking History [*F*(1,87) = 3.88, *p* = 0.052, η^2^p = 0.043] and Age x Drinking History [*F*(1,87) = 2.87, *p* = 0.094, η^2^p = 0.032] interactions were detected. As illustrated in Figure 5L, male binge-drinking mice exhibited fewer immobile episodes than their water controls (*p* = 0.022, *d* = 0.674), while female binge-drinking mice exhibited more immobile episodes than their male binge-drinking counterparts (*p* = 0.001, *d* = 0.973). As illustrated in Figure 5M, the Age x Drinking History interaction revealed fewer immobile episodes by the adolescent binge-drinking mice versus their water controls (*p* = 0.037, *d* = 0.618) and the adolescent water control mice also exhibited more immobile episodes than their adult counterparts (*p* = 0.051, *d* = 0.572). No other significant interactions were observed (3-way ANOVA: *p* = 0.773, η^2^p = 0.001).

### Morris water maze

#### Flag test

Sex × Age × Drinking History ANOVAs failed to detect any significant interactions or main effects for the time taken to locate the flagged platform during either early [all *p’s* > 0.582] or later withdrawal [all *p’s* > 0.343]. The data are presented in Table 5 and indicate comparable visual and swimming ability across our different experimental groups prior to maze training. These findings also indicate that group differences in the Porsolt swim test, conducted 1–2 days prior (Figure 5), did not carry over to the Morris water maze.

**TABLE 5:** Summary of the negative results for the time taken (in sec) to locate the flagged platform in the Morris water maze.

	Early withdrawal		Late withdrawal	
	**Females**	**Males**	**Females**	**Males**
Adolescent-H2O	70.39 ± 13.60 *n* = 12	70.32 ± 12.16 *n* = 12	53.35 ± 13.20 *n* = 12	46. 62 ± 11.39 *n* = 12
Adolescent-EtOH	67.89 ± 13.18 *n* = 12	57.23 ± 12.35 *n* = 12	54.31 ± 13.32 *n* = 12	59.58 ± 13.12 *n* = 12
Adult-H2O	74.84 ± 10.89 *n* = 16	63. 76 ± 12.52 *n* = 12	53.37 ± 13.48 *n* = 12	69.49 ± 14.17 *n* = 12
Adult-EtOH	68.45 ± 12.41 *n* = 8	65.75 ± 13.17 *n* = 12	38.28 ± 9.50 *n* = 12	54.65 ± 11.98 *n* = 12

The data represent the means ± SEMs for the number of mice indicated.

#### Morris maze acquisition

**FIGURE 6:**
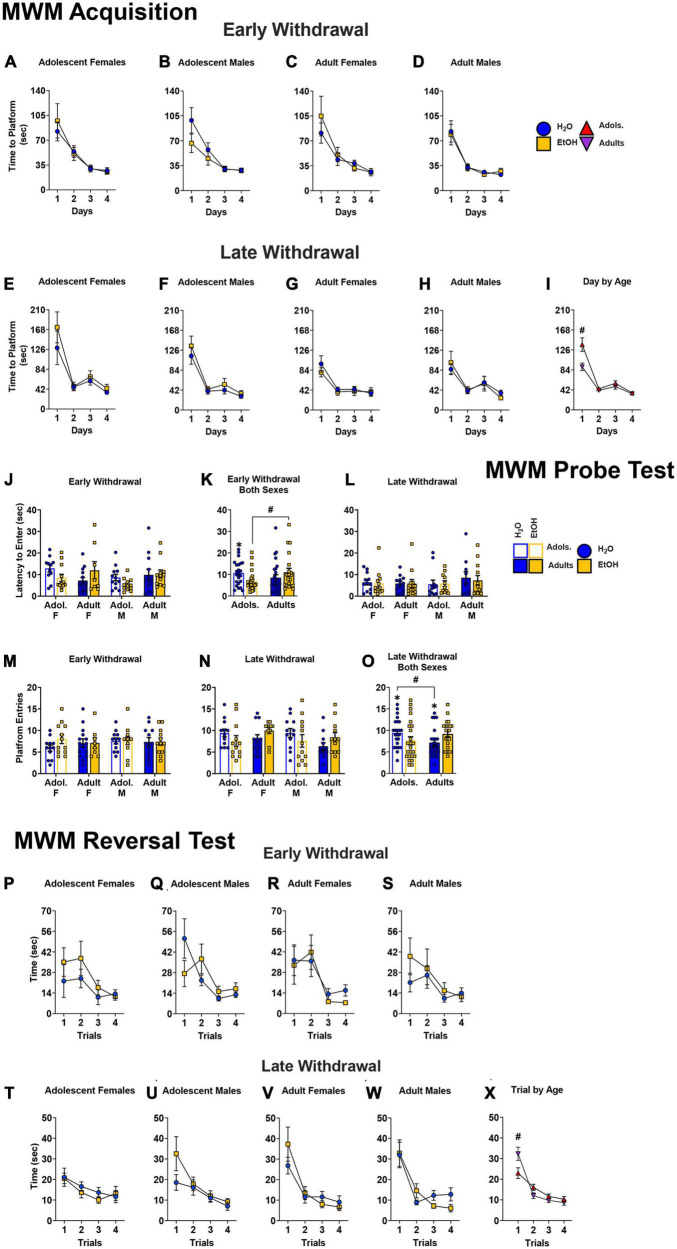
Depiction of the results of the Day × Sex × Age × Drinking History mixed-model ANOVAs evaluating spatial learning during the different phases of testing in the Morris water maze. **(A–D)** No group differences were noted for the average time taken by mice tested in early alcohol withdrawal to locate the hidden platform during Morris maze acquisition. The sample sizes for mice tested on WD1 are the following: **(A)** H2O (*n* = 9), EtOH (*n* = 12); **(B)** H2O (*n* = 11), EtOH (*n* = 12); **(C)** H2O (*n* = 15), EtOH (*n* = 8); **(D)** H2O (*n* = 12), EtOH (*n* = 12) **(E–H)** For mice trained during later withdrawal, we detected no significant Day by Sex by Age by Drinking History interaction. The sample sizes for mice tested on WD are the following); **(E)** H2O (*n* = 12), EtOH (*n* = 11); **(F)** H2O (*n* = 12), EtOH (*n* = 9); **(G)** H2O (*n* = 12), EtOH (*n* = 12); **(H)** H2O (*n* = 12), EtOH (*n* = 11). **(I)** However, a significant Day by Age interaction was detected that reflected a longer time taken by adolescent versus adult mice to locate the platform on the first day training [adolescents (*n* = 44), adults (*n* = 47)]. **(J)** When tested in early alcohol withdrawal, no significant 3-way interaction was detected for the latency to enter the platform’s former location [females: adolescent H2O (*n* = 9); adolescent EtOH (*n* = 12); adult H2O (*n* = 11); adult EtOH (*n* = 12); males: adolescent H2O (*n* = 15); adolescent EtOH (*n* = 8); adult H2O (*n* = 12); adult EtOH (*n* = 12)]. **(K)** On the probe test, an Age by Drinking History interaction indicated that adolescent-onset mice exhibited a shorter latency to enter the platform’s former location in the NE quadrant compared to their age-matched water control counterparts and the adult-onset mice [adolescents: H2O (*n* = 24); EtOH (*n* = 20); adults: H2O (*n* = 23); EtOH (*n* = 24). **(L)** No significant group differences were found for this measure in mice tested in later withdrawal [females: adolescent H2O (*n* = 12); adolescent EtOH (*n* = 11); adult H2O (*n* = 12); adult EtOH (*n* = 19); males: adolescent H2O (*n* = 12); adolescent EtOH (*n* = 12); adult H2O (*n* = 12); adult EtOH (*n* = 12)]. **(M)** We also did not detect group differences on WD1 with regards of the number of entries to the former site of the platform [samples sizes same as panel **(A)**]. **(N)** However, on WD30, a significant Age by Drinking History interaction was observed [sample sizes same as Panel **(C)**]. **(O)** This interaction reflected trends for more entries by adolescent EtOH versus adolescent H2O mice, as well as fewer entries by adult EtOH versus adult H2O mice. Additionally, adolescent H2O mice made significantly more entries than adult H2O mice [adolescents (*n* = 44), adults (*n* = 47)]. For the data from the reversal learning phase of the study, Trial by Sex by Age by Drinking History ANOVAs revealed no significant group differences for the time taken to locate the repositioned platform during the reversal test when mice were tested in either early **(P–S)** or late withdrawal **(T–W)**. Sample sizes are the following: **(P)** H2O (*n* = 10), EtOH (*n* = 12); **(Q)** H2O (*n* = 12), EtOH (*n* = 12); **(R)** H2O (*n* = 15), EtOH (*n* = 8); **(S)** H2O (*n* = 11), EtOH (*n* = 11); **(T)** H2O (*n* = 12), EtOH (*n* = 10); **(U)** H2O (*n* = 11), EtOH (*n* = 10); **(V)** H2O (*n* = 11), EtOH (*n* = 12); **(W)** H2O (*n* = 12), EtOH (*n* = 11). **(X)** However, a significant Trial by Age interaction was observed for the mice tested in late withdrawal that reflected a longer latency of adult-onset versus adolescent-onset mice to locate the repositioned platform on the first reversal trial [adolescents (*n* = 43), adults (*n* = 46)]. The data represent the means ± SEMs for the number of mice indicated above. ^#^*p* < 0.05, adolescents vs. adults.

No significant Day × Sex × Age × Drinking History interaction was noted for the time taken to locate the hidden platform across the 4 days of the Morris maze acquisition for the mice tested in early withdrawal (4-way ANOVA: *p* = 0.865, ηp^2^ = 0.001). As depicted in Figures 6A–D, all mice successfully acquired the maze as indicated by a main Day effect [*F*(1.49, 123.79) = 65.95, *p* < 0.001, η^2^p = 0.443; all other p’s > 0.118]. We also detected no significant Day × Sex × Age × Drinking History interaction for the time taken to complete the Morris maze by mice tested in later withdrawal [Figures 6E–H; 4-way ANOVA: *p* = 0.464, η^2^p = 0.008]. However, a significant Day × Age interaction was detected in later withdrawal [*F*(1.41, 116.64) = 5.83, *p* = 0.009, η^2^p = 0.066]. As illustrated in Figure 6I, this interaction reflected more time taken by adolescent-onset versus adult-onset mice to locate the hidden platform on the first day of training, irrespective of their sex or alcohol-drinking history (*p* = 0.004).

#### Probe test

The data for the latency to enter the platform’s former location on WD1 are presented in Figure 6J. Analyses of a Sex × Age × Drinking History ANOVA for the mice tested in early withdrawal failed to detect a significant 3-way interaction for the latency to first enter the platform’s former location (*p* = 0.333, η^2^p = 0.011); however, a Age x Drinking History interaction was detected for this variable [*F*(1,85) = 5.50, *p* = 0.021, η^2^p = 0.061]. This interaction reflected a shorter latency to first enter the platform location by adolescent-onset mice relative to their age-matched water-drinking counterparts (*p* = 0.047, *d* = 0.589), and to the adult binge-drinking mice (Figure 6K; *p* = 0.036, *d* = 0.651). For the mice tested in later withdrawal, no significant main effects or interactions were found with respect to this variable [Figure 6L; Sex × Age × Drinking History ANOVA: *p* = 0.703, η^2^p = 0.002, all other *p’s* > 0.306].

As alternate indices of spatial recall, we also examined the number of entries into the platform’s former location. No significant main effects or interactions were observed for the number of entries into the platform’s former location for mice tested in early withdrawal [Figure 6M; 3-way ANOVA: *p* = 0.444, η^2^p = 0.007; all other *p’s* > 0.386]. However, a significant Age x Drinking History interaction was observed for the number of former platform entries for the mice tested in later withdrawal [Figure 6N; *F*(1,87) = 6.63, *p* = 0.012, η^2^p = 0.071]. This interaction reflected a trend for more entries by adolescent-onset water controls versus their binge-drinking counterparts, with a medium effect size (Figures 6O, F, left; *p* = 0.087, *d* = 0.500), with a similarly sized, but opposite, group difference was noted for the adult-onset mice (Figure 6O, right; *p* = 0.060, *d* = 0.557). Lastly, adolescent-onset water-drinking controls made more entries, overall, than their adult-onset counterparts (Figure 6O; *p* = 0.036, *d* = 0.614).

#### Reversal test

For the mice tested in early withdrawal (Figures 6P–S), a Trial × Sex × Age × Drinking History ANOVA revealed no significant group differences for the time taken to locate the repositioned platform during the reversal test [all ANOVAs *p’s* > 0.158]. In contrast, a significant Trial x Age interaction was detected for the mice tested in later withdrawal (Figures 6T–W) [*F*(1.88, 152.49) = 5.66, *p* = 0.001, η^2^p = 0.065] that reflected a longer time taken to find the repositioned platform by adult-onset versus adolescent-onset mice on the initial reversal trial (Figure 6X; Trial 1: *p* = 0.034). No other significant interactions were observed between the binge-drinking and water-drinking groups, however, a main effect of Trial illustrated a progressive reduction in the time required to locate the platform [Trial Effect: *F*(1.88,152.49) = 46.07, *p* < 0.001, η^2^p = 0.363].

### Radial arm water maze

#### Number of reference memory errors

**FIGURE 7:**
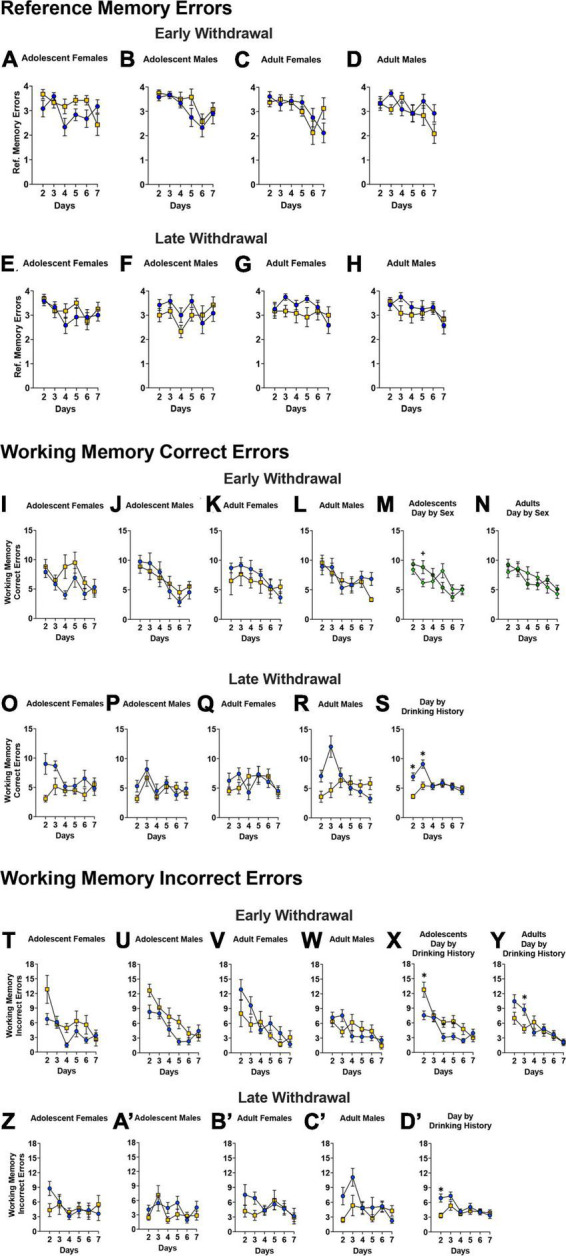
Depiction of the results of the Day × Sex × Age × Drinking History mixed-model ANOVAs evaluating reference memory, working memory correct and incorrect errors in the Radial Arm Maze. **(A–D)** For mice tested in early withdrawal (top), a significant Day by Sex by Age by Drinking History interaction was detected for the number of reference memory errors during the first week of radial arm maze training testing. **(A,C)** In female mice, a significant Day by Age by Drinking History interaction was found and follow up analyses indicated a significant Day by Drinking History interaction for adolescent females. However, no statistically significant drinking history differences were noted for the adolescent females on any of the training days [females: adolescent H20 (*n* = 12); adolescent EtOH (*n* = 12); adult H2O (*n* = 16); adult EtOH (n = 8)]. **(B,D)** For male mice, a Day by Age interaction was observed, reflecting more errors by adult versus adolescent males on day 6 of training only irrespective of drinking history [males: adolescent H2O (*n* = 12); adolescent EtOH (*n* = 12); adult H2O (*n* = 12); adult EtOH (*n* = 12)]. **(E–H)** For the mice tested in later withdrawal, a significant Day by Age interaction was found on day 4 of training, with more errors by adults than adolescents. For WD30, sample sizes were the following: **(E)** H2O (*n* = 12), EtOH (*n* = 12); **(F)** H2O (*n* = 12), EtOH (*n* = 12); **(G)** H2O (*n* = 12), EtOH (*n* = 12); **(H)** H2O (*n* = 12), EtOH (*n* = 12). Note that interactions that do not include Drinking History as a factor have not been included in panels **(A–H)**. **(I–L)** For mice tested in early withdrawal, there was a significant Day by Sex by Age by Drinking History interaction for working memory correct errors committed in the radial arm maze. The samples sizes are the same as panels **(A–D)**. When collapsed along the Age factor, **(M)** a significant Day by Sex interaction for adolescent mice indicated that males committed more errors on day 3 of training [females (*n* = 24), males (*n* = 24)]. **(N)** However, only a main effect of Day was observe for adult mice [females (*n* = 24), males (*n* = 24)]. **(O–S)** For WD30 mice, no significant 4-way interaction was found. The samples sizes are the same as panels **(E–H)**. **(S)** There was a significant Day by Drinking History interaction during late withdrawal that indicated binge-drinking mice committed fewer errors on the first two days [H2O (*n* = 48), EtOH (*n* = 48)]. **(T–W)** No significant Day by Sex by Age by Drinking History interaction was detected for the number of working memory incorrect errors committed by the mice tested in early withdrawal. The sample sizes are the same as panels **(A–D)**. However, deconstruction of the significant Day by Age by Drinking History interaction along the Age factor indicated that **(X)** adolescent-onset binge-drinking mice made more errors on certain days [H2O (*n* = 24), EtOH (*n* = 24)], while **(Y)** adult-onset binge-drinking mice committed fewer errors only on day 3 [H2O (*n* = 28), EtOH (*n* = 20)]. **(Z–C’)** For the mice tested in later withdrawal, no significant Day by Age by Sex by Drinking History interaction was found. The sample sizes are the same as panels **(E–H)**. **(D’)** However, a significant Day by Drinking History interaction was detected that reflected a progressive decline in working memory incorrect errors in water-drinking animals versus the relatively flat time-course of errors exhibited by binge-drinking mice [H2O (*n* = 48), EtOH (*n* = 48)]. The data represent the means ± SEMs of the number of mice indicated above. *p < 0.05, EtOH vs. H2O; ^+^*p* < 0.05, Female vs. Male.

For the mice tested in early withdrawal, a significant Day × Sex × Age × Drinking History interaction was detected for the number of reference memory errors during the first week of radial arm maze training (Figures 7A–D) [*F*(4.32, 380.37) = 3.27, *p* = 0.010, η^2^p = 0.036]. This 4-way interaction was first analyzed along the Sex factor and indicated a significant Day × Age × Drinking History interaction for the female mice [*F*(4.20, 184.67) = 4.00, *p* = 0.003, η^2^p = 0.083]. The Day x Age x Drinking History interaction observed in female mice was further deconstructed along the Age factor and indicated a significant Day × Drinking History interaction for the adolescent-onset females [*F*(3.93, 86.46) = 3.03, *p* = 0.022, η^2^p = 0.121]. However, while it appeared that adolescent-onset binge-drinking females committed more reference memory errors than their water-drinking counterparts on several days during this initial training (Figure 7A), *post-hoc* tests did not indicate any statistically significant water-alcohol differences (all *p’s* > 0.072). The comparable follow-up analysis of the significant Day × Age × Drinking History interaction for adult-onset females indicated only a significant main effect of Day (Figure 7C) [*F*(3.69,127.17) = 3.76, *p* = 0.009, η^2^p = 0.146]. Thus, a prior history of binge-drinking during adulthood did not influence reference memory in adult females tested during early alcohol withdrawal. For the males tested in early withdrawal, no significant Day × Age × Drinking History interaction was found upon deconstruction of the significant 4-way interaction along the Age factor [ANOVA: *p* = 0.524, η^2^p = 0.019]. However, a Day x Age interaction was observed [*F*(4.00, 175.76) = 2.18, *p* = 0.074, η^2^p = 0.047], that reflected a trend toward more reference memory errors committed by adult versus adolescent males on day 6 of training only (Figures 7B, D; *p* = 0.061).

For mice tested in later withdrawal (Figures 7E–H), a significant Day × Age interaction [*F*(5, 440) = 2.72, *p* = 0.020, η^2^p = 0.030] was detected. However, *post hoc* analyses indicated that this interaction reflected more reference memory errors committed by adults vs. adolescents only on day 4 of training (*p* = 0.041) and thus, this interaction is not depicted.

#### Working memory correct errors

Analyses of the data from the mice tested in early withdrawal identified a significant Day × Sex × Age × Drinking History interaction for the number of working memory correct errors during the first week of testing (Figures 7I–L) [*F*(4.48,394.13) = 2.43, *p* = 0.041, η^2^p = 0.027]. While deconstruction along the Sex factor indicated no significant interactions [ANOVA for females, *p’s* > 0.212; ANOVA for males, *p’s* > 0.162], deconstruction along the Age factor revealed a significant Day x Sex interaction for the adolescent-onset mice [*F*(4.31,189.61) = 2.76, *p* = 0.026, η^2^p = 0.059], that reflected a greater number working memory correct errors in males versus females only on day 3 of radial arm maze training (Figure 7M; *p* = 0.044, all other *p*’s > 0.065). In contrast, no interactions were detected in adult-onset mice, with all mice exhibiting a progressive reduction in working memory correct errors with training (Figure 7N) [Day effect: *F*(4.24,186.56) = 4.89, *p* < 0.001, η^2^p = 0.100].

Analyses of the data from mice tested in later withdrawal failed to indicate a significant 4-way interaction [Figures 7O–R; Day × Sex × Age × Drinking History ANOVA: *p* = 0.168, η^2^p = 0.064]. However, a significant Day x Drinking History interaction was detected [*F*(4.429,389.719) = 6.02, *p* < 0.001 η^2^p = 0.064] that reflected *fewer* working memory correct errors committed by binge- versus water-drinking mice on the first two days of radial arm maze training (Figure 7S; *p* < 0.001)–a result suggestive of better working memory performance in binge- versus water-drinking mice. However, it is notable that the time-course of working memory errors committed by binge-drinking mice during later withdrawal was relatively flat (Figure 7S); in fact, binge-drinking mice committed significantly *more* working memory correct errors later during training than at the start of training (Figure 7S; day 2 vs. days 3–5, all *p*’s < 0.027). In contrast, the number of working memory correct errors committed by water-drinking mice declined progressively over the course of training, indicative of intact learning (Figure 7S; day 2 vs. subsequent days, all *p’s* < 0.046).

#### Working memory incorrect errors

No significant Day × Sex × Age × Drinking History interaction was detected for the number of working memory incorrect errors committed by the mice tested in early withdrawal (Figures 7T–W; 4-way ANOVA: *p* = 0.588, η^2^p = 0.008). However, a significant Day × Age x Drinking History interaction was found for this time-point [*F*(4.26,374.68) = 2.76, *p* = 0.018, η^2^p = 0.030]. Deconstruction of this interaction along the Age factor indicated a significant Day x Drinking History interaction for both age groups [ANOVA for adolescent-onset: *F*(4.31,198.12) = 2.84, *p* = 0.017, η^2^p = 0.058; ANOVA for adult-onset: *F*(4.03,185.41) = 2.91, *p* = 0.014, η^2^p = 0.060]. On days 2, 4, and 5, adolescent-onset binge-drinking mice made more working memory incorrect errors versus their water controls (Figure 7X; Day 2: *p* = 0.005; Day 4: *p* = 0.023; Day 5: *p* = 0.034). In contrast, adult-onset binge-drinking mice committed fewer working memory incorrect errors than water controls but only on day 3 (Figure 7Y; *p* = 0.014). As depicted in Figures 7X, Y, the number of working memory incorrect errors declined progressively in both water- and binge-drinking mice, indicative of learning in all groups when tested at the earlier time-point.

For the mice tested in later withdrawal, no significant Day × Age × Sex × Drinking History interaction was found for the number of working memory incorrect errors [Figures 7Z–C’; 4-way ANOVA *p* = 0.267, η^2^p = 0.014]. However, a significant Day x Drinking History interaction [*F*(4.16,365.85) = 2.68, *p* = 0.030, η^2^p = 0.029] was detected that reflected a lower number of working memory incorrect errors in binge- versus water-drinking mice, but only on day 2 of training (Figure 7D’; *p* < 0.001, all other *p*’s > 0.092). Consistent with the data for the number of working memory correct errors, water-drinking controls tested in later withdrawal exhibited a progressive decline in the number of working memory incorrect errors with training (Figure 7D’; days 2 and 3 vs. 4–7, all *p’s* < 0.041), while the time-course of behavior was flat in binge-drinking animals (Figure 7D’; day 2 < day3, *p* = 0.032), indicative of little to no learning.

#### Chaining behavior

**FIGURE 8:**
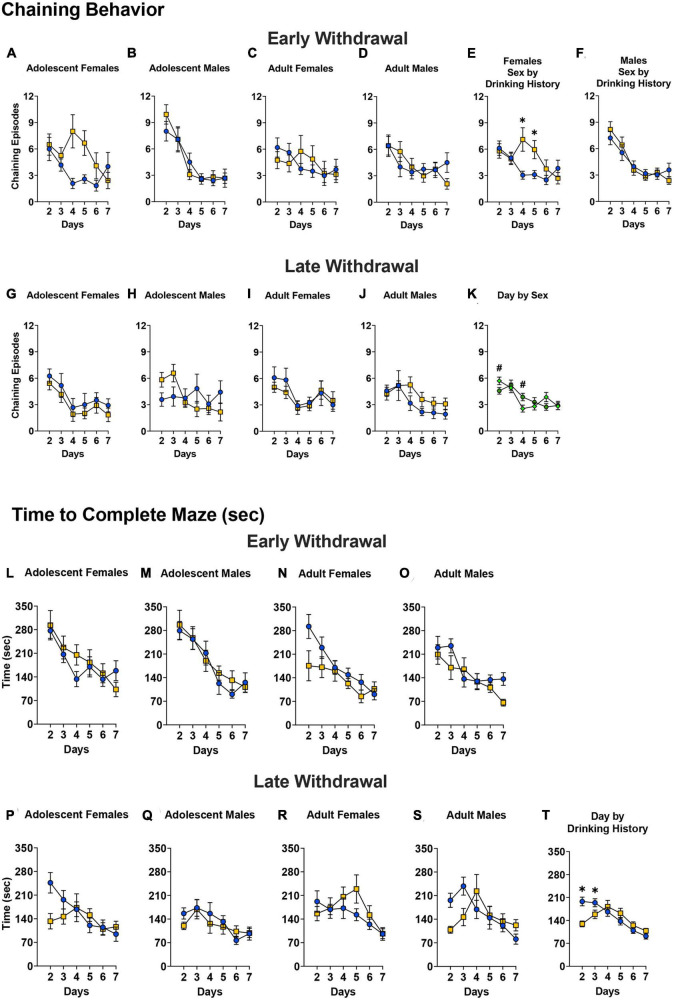
Depiction of the results of the Day × Sex × Age × Drinking History mixed-model ANOVAs evaluating non-spatial navigation (chaining episodes) and time taken to navigate the Radial Arm Maze. **(A–D)** No significant 4-way interaction in mice tested in early withdrawal. For early withdrawal, the sample sizes were as follows: **(A)** H2O (*n* = 12), EtOH (*n* = 12); **(B)** H2O (*n* = 12), EtOH (*n* = 12); **(C)** H2O (*n* = 16), EtOH (*n* = 8); **(D)** H2O (*n* = 12), EtOH (*n* = 12). **(E)** However, a significant Day by Sex by Drinking History interaction was detected that reflected more chaining behavior by binge-drinking females than water controls on days 4 and 5 [H2O (*n* = 28), EtOH (n = 20)]. **(F)** No significant interaction was detected for males tested in early withdrawal [H2O (*n* = 24), EtOH (*n* = 24)]. **(G–J)** In mice tested in later withdrawal, no significant 4-way interaction was found. For late withdrawal, the sample sizes were as follows: **(G)** H2O (*n* = 12), EtOH (*n* = 12); **(H)** H2O (*n* = 12), EtOH (*n* = 12); **(I)** H2O (*n* = 12), EtOH (*n* = 12); **(J)** H2O (*n* = 12), EtOH (*n* = 12). **(K)** However, a significant Day by Sex interaction was detected that reflected sex differences in chaining on day 2 and 4 of training [females (*n* = 48), males (*n* = 48)]. The data represent the means ± SEMs for the number of mice indicated above. **(L–O)** For the mice tested in early withdrawal, there were no significant Day by Sex by Age by Drinking History interactions on the total time to complete the maze, and all mice showed improvement in maze completion over time. The sample sizes are the same as for panels **(A–D)**. **(P–S)** For the mice tested in later withdrawal, no significant 4-way interaction was detected. The sample sizes are the same as for panels **(G–J)**. **(T)** However, a significant Day by Group interaction was noted, which reflected a shorter latency to complete the maze on days 2 and 3 by binge-drinking mice [H2O (*n* = 48), EtOH (*n* = 48)]. The data represent the means ± SEMs for the number of mice indicated above. *p < 0.05, EtOH vs. H2O; ^#^*p* < 0.05, adolescents vs. adults.

The Day × Sex × Age × Drinking History ANOVA for the mice tested in early withdrawal indicated in no significant four-way interaction for chaining behavior [Figures 8A–D; 4-way ANOVA: *p* = 0.184, η^2^p = 0.017]. However, a significant Day × Sex × Drinking History interaction was observed [*F*(4.26,374.80) = 3.02, *p* = 0.016, η^2^p = 0.033]. Deconstruction of this interaction along the Sex factor yielded a significant Day × Drinking History interaction for the female mice (Figure 8E) [*F*(3.69,169.50) = 3.96, *p* = 0.005, η^2^p = 0.079]. As illustrated (Figure 8E), binge-drinking females exhibited more chaining behavior than their water controls on day 4 (*p* = 0.002) and day 5 (*p* = 0.008) of training. No significant interactions were detected for the male mice tested in early withdrawal (Figure 8F) [ANOVA: *p* = 0.416, η^2^p = 0.021]. As illustrated (Figure 8F), all males exhibited a training-dependent reduction in the amount of chaining behavior [Day effect: *F*(4.01,184.40) = 21.63, *p* < 0.001, η^2^p = 0.320; *post-hoc* tests, days 2 and 3 versus days 4–7, all *p’s* < 0.010].

The analyses of the data for the mice tested in later withdrawal failed to detect a significant Day × Age × Sex × Drinking History interaction [Figures 8G–J; 4-way ANOVA, *p* = 0.338, η^2^p = 0.010]. However, a significant Day x Sex interaction was observed [*F*(4.16,365.77) = 2.57, *p* = 0.036, η^2^p = 0.028] that reflected more chaining episodes in females versus males on day 2 of training, while males exhibited more chaining episodes on day 4 [Figure 8K; Day 2: *p* = 0.053; Day 4: *p* = 0.026]. As illustrated in Figure 8K, male mice exhibited a progressive decline in the amount of chaining across the first week of testing, indicative of a shift from non-spatial to spatial learning strategies [Day 2 vs., Days 4 –6: *p*’s < 0.033]. While chaining behavior declined early during training in the females tested in later withdrawal (Figure 8K; days 2 and 3 vs. days 5–7; all *p’s* < 0.003), this behavior plateaued, with females exhibiting more chaining on day 6, relative to day 4 (*p* = 0.032) and day 7 (Figure 8K; *p* = 0.037).

#### Time to complete the maze

No significant interactions between Day × Sex × Age × Drinking History were detected for the total time taken to find all the platforms in the radial arm maze when the mice were tested in early withdrawal [Figures 8L–O); all ANOVA *p’s* > 0.147]. All mice exhibited a progressive decline in the amount of time taken to complete the maze [Day effect: *F*(4.15, 364.98) = 42.03, *p* < 0.001, η^2^p = 0.323; *post-hoc* tests for all groups, all *p’s* < 0.030].

No significant 4-way interaction was observed with respect to the time taken by mice to complete the radial arm maze during later withdrawal [Figures 8P–S; Day × Sex × Age × Drinking History ANOVA: *p* = 0.206, η^2^p = 0.018). However, a significant Day x Group interaction [*F*(4.08, 358.22) = 4.96, *p* = 0.001, η^2^p = 0.053] was found that reflected a shorter time taken by binge- versus water-drinking mice on days 2 and 3 of training [Figure 8T; Day 2 p < 0.001; Day 3 *p* = 0.052). As illustrated in Figure 8T, the WD30 water-drinking mice exhibited a progressive decline in the time taken to complete the maze, consistent with learning (day 2 vs. days 5–7; all *p’s* < 0.002). In contrast, the time-course for this variable exhibited an inverted U-shape in the binge-drinking mice tested in later withdrawal, with the longest latency to complete the maze observed on day 4 of training (Figure 8T; all *p’s* < 0.043).

### Replicate testing for alcohol withdrawal-induced negative affect

**FIGURE 9:**
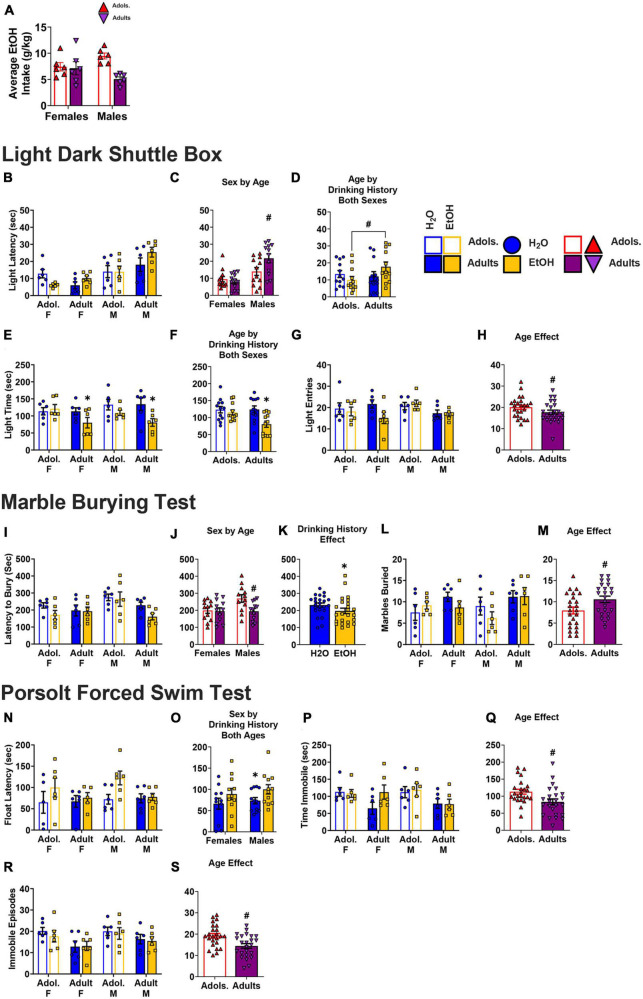
Depiction of the results from Study 2. **(A)** In the replicate study, a significant age x sex interaction was detected for the amount of alcohol consumed (*n* = 6/sex/age), that reflected more alcohol intake by adolescent versus adult males. **(B)** Summary of the data for the latency to first enter the light-side of the light-dark shuttle box on WD 1 (*n* = 6/sex/age/drinking history). The ANOVA conducted on this variable revealed a Sex by Age interaction [**(C)**; *n* = 12/sex/drinking history] and an Age by Drinking History interaction [**(D)**; *n* = 12/age/drinking history]. **(E)** Summary of the data for the time spent in the light side (in sec), highlighting a main drinking history effect in adult mice (*n* = 6/sex/age/drinking history). **(F)** The ANOVA conducted on this variable indicated also an Age × Drinking History interaction that reflected H2O-EtOH differences in adult mice (*n* = 12/age/drinking history). **(G)** Summary of the number of entries into the light side of the shuttle box (*n* = 6/sex/age/drinking history). **(H)** The ANOVA indicated fewer light-side entries by adults vs. adolescents (*n* = 24/age). **(I)** Summary of the data for the latency to begin marble-burying (*n* = 6/sex/age/drinking history). The ANOVA indicated a Sex × Age interaction [**(J)**; *n* = 12/sex/age], as well as a main Drinking History effect [**(K)**; *n* = 24/drinking history]. **(L)** Summary of the data for the number of marbles buried (*n* = 6/sex/age/drinking history). **(M)** The ANOVA indicated that adults buried more marbles than adolescent mice (*n* = 24/age). **(N)** Summary of the data for the latency to first floatin in the forced swim test (*n* = 6/sex/age/drinking history) [females: adolescent-H20 (*n* = 5); adolescent-EtOH (*n* = 6); adult-H2O (*n* = 6); adult-EtOH (*n* = 5); males: *n* = 6/age/drinking history]. **(O)** The ANOVA revealed a Sex × Drinking History inaction, but no specific H2O-EtOH differences were detected [female-H2O (*n* = 11); female-EtOH (*n* = 11); male-H2O (*n* = 12); male-EtOH (*n* = 12]. **(P)** Summary of the data for the time spent immobile (*n* = 6/sex/age/drinking history). **(Q)** The ANOVA indicated less time immobile in adult versus adolescent mice (*n* = 24/age). **(R)** Summary of the data for the number of immobile episodes (*n* = 6/sex/age/drinking history), the ANOVA for which indicated fewer immobile episodes in adult versus adolescent mice [**(S)**; *n* = 24/age]. The data are presented as the means ± SEMs for the respective number of mice indicated above. *p < 0.05 H2O vs. EtOH; ^#^*p* < 0.05, adolescents vs. adults (main Age effect).

An analysis of the average total alcohol consumed over the 2-week drinking period indicated a significant Sex × Age interaction [*F*(1,23) = 6.33, *p* = 0.021; η^2^p = 0.240]. In this replicate study, the interaction reflected higher alcohol intake by male adolescent mice versus their adult controls [*t*(10) = 6.28, *p* < 0.001], with no age difference detected for the relatively high alcohol intake exhibited by female subjects (Figure 9A; *t*-test, *p* = 0.858).

### Light-dark shuttle box

#### Latency to enter the light-side

Under these more insulated testing conditions, we detected two significant interactions with respect to the latency to first enter the light-side of the light-dark shuttle box (Figure 9B). As illustrated in Figure 9C, a Sex × Age interaction [*F*(1,47) = 5.57, *p* = 0.023; η^2^p = 0.122] reflected a shorter latency of adolescent versus adult males to enter the light-side [*t*(22) = 2.24, *p* = 0.035], with no age difference observed in females (*t*-test, *p* = 0.516). We also detected a significant Age X Drinking interaction for this variable (Figure 9D) [*F*(1,47) = 5.59, *p* = 0.023; η^2^p = 0.123]. Although inspection of Figure 9D suggested that this interaction reflected specifically an alcohol-induced increase in the latency of adult mice to first enter the light-side, water-alcohol differences were not detected for either age group (*t*-tests, *p*’s > 0.158). Rather, the Age × Drinking interaction reflected a longer latency to enter the light side by alcohol-experienced adults versus their adolescent counterparts [*t*(22) = 2.28, *p* = 0.032], with no age difference noted for water controls (Figure 9D; *t*-test, *p* = 0.697).

#### Light side time and entries

A summary of the data for the time spent on the light side is depicted in Figure 9E. A significant Age × Drinking interaction was also detected for this variable [*F*(1,47) = 3.63, *p* = 0.064; η^2^p = 0.083], which reflected less time spent by alcohol-experienced adults versus their water controls [*t*(22) = 3.14, *p* = 0.055] with no water-alcohol differences detected in adolescent mice (Figure 9F; *t*-test, *p* = 0.499). Although it appeared that adult female alcohol-experienced mice entered the light-side fewer times than their water controls (Figure 9G), we detected only an overall effect of age with respect to this variable, with adults spending less time in the light-side than adolescents (Figure 9H) [Age effect: *F*(1,47) = 2.93, *p* = 0.095; η^2^p = 0.068; other *p*’s > 0.133; η^2^p’s < 0.056].

### Marble-burying

#### Latency to bury

A summary of the data for the latency to begin marble burying is provided in Figure 9I. Under the more insulated testing conditions, we detected a significant Age × Sex interaction for the latency to begin marble burying [*F*(1,47) = 3.68, *p* = 0.062; η^2^p = 0.084] that reflected a longer latency of adolescent versus adult males [*t*(22) = 2.71, *p* = 0.013], with no age difference noted for females (Figure 9J; *t*-test, *p* = 0.885). We also detected an overall Drinking effect [*F*(1,47) = 3.46, *p* = 0.070; η^2^p = 0.080] that reflected a shorter latency to bury in alcohol-experienced mice versus water controls, irrespective of the animals’ age or sex (Figure 9K; Drinking interactions, all *p*’s > 0.149; η^2^p’s < 0.052).

#### Marbles buried

In contrast, we detected only an overall Age effect with respect to the number of marbles buried (Figure 9L) [*F*(1,47) = 5.482, *p* = 0.024; η^2^p = 0.121; other *p*’s > 0.117; η^2^p’s < 0.061], that reflected more marbles buried by adult versus adolescent mice (Figure 9M).

### Forced swim test

#### Latency to first immobile episode

As depicted in Figure 9N, there was considerable variability in the latency to first float in the forced swim test even when extreme outliers were removed. However, we did detect a significant Sex × Drinking History interaction (Figure 9O) [*F*(1,45) = 70.15, *p* = 0.050; η^2^p = 0.825]. This interaction reflected a longer latency to float by alcohol-experienced males versus their water controls [*t*(22) = 2.022, *p* = 0.056], with no significant alcohol-water difference detected in females (*t*-test, *p* = 0.235). No Age effect or interactions were detected for this variable (*p*’s > 0.208; η^2^p < 0.650).

#### Number and duration of immobility

In contrast to the latency to float, we detected no alcohol or sex effects for the time spent floating (Figure 9P; *p*’s > 0.187; η^2^p < 0.044) or the number of floating episodes (Figure 9R; *p*’s > 0.282; η^2^p < 0.029) or in the forced swim test. Instead, we detected only main Age effects for both variables (Figures 9O, S) [for float episodes, Age effect: *F*(1,47) = 8.73, *p* = 0.005; η^2^p = 0.179; for float time (sec), Age effect: *F*(1,47) = 6.86, *p* = 0.012; η^2^p = 0.146] that reflected less floating-related behavior in adult vs. adolescent mice.

## Discussion

The present study was designed to expand upon a recent report from our group describing weak interactions between a sub-chronic (i.e., 2 week) history of binge-drinking, the age of drinking-onset and sex in the affective consequences of alcohol assayed at 1 versus 70 days withdrawal (Jimenez Chavez et al., 2020). The results of this prior study (Jimenez Chavez et al., 2020) contrasted with earlier reports of robust, age-dependent, effects in the marble-burying, light-dark box and forced swim tests (Lee et al., 2016, 2017a,b, 2018a,b; Szumlinski et al., 2019). As these latter studies employed a single sex and tested for negative affect at 1 versus 30 days withdrawal, herein, we segregated the testing of our male and female mice on WD1 and WD30 to reduce the influence of chemosensory social stimuli from the opposite sex on behavior. Based on a recent study of older mice (>6 months of age) indicating sex differences in alcohol-induced cognitive impairment (Jimenez Chavez et al., 2022), as well as published work from other groups indicating that a history of alcohol-drinking during adolescence can accelerate the onset of cognitive decline (e.g., Ledesma et al., 2021; Van Hees et al., 2022), we also tested for interactions between our subject factors with respect to spatial learning and memory in the Morris water maze, as well as reference and working memory in the radial arm maze. Although we detected some affective and cognitive effects of binge-drinking, the group differences were not as robust as in prior work when a single sex was tested. Thus, we also conducted an additional study to best mimic the procedural conditions of our prior work (i.e., Lee et al., 2016, 2017b, 2018a), in which we single-housed water controls during drinking procedures and behavioral testing was conducted in series in distinct procedural rooms.

### Robust binge-drinking for 2 weeks elicits relatively few effects on negative affect during alcohol withdrawal

A summary of the effects of alcohol withdrawal on our behavioral measures from our two studies is presented in Table 6. As expected (Finn et al., 2010; Strong et al., 2010; Wilsnack et al., 2018; Szumlinski et al., 2019; Jimenez Chavez et al., 2020, 2022), the female mice in the larger study binge-drank more alcohol than males and exhibited higher BACs (Figure 2B). Also as expected (Moore et al., 2010; Melón et al., 2013; Lee et al., 2016, 2017b, 2018a; Szumlinski et al., 2019; Jimenez Chavez et al., 2020), adolescents consumed more alcohol and attained higher BACs than their adult counterparts (Figure 2A). Moreover, BACs on the day of sampling were at or above the NIAAA 80 mg/dL criterion for binge-drinking (National Institute on Alcohol Abuse and Alcoholism [NIAAA], 2004) and BACs correlated with alcohol intake, with adult males exhibiting the lowest intakes/BACs, and adolescent females exhibiting the highest intakes/BACs (Figure 2C). However, in the smaller scale study (*n* = 6/sex/age/group), the sex and age differences were less robust, owing to the relatively high alcohol intake of the adolescent males (Figure 9A).

**TABLE 6:** Summary of the effects of a 2-week history of binge-drinking upon our measures of negative affect and cognition.

Dependent variable	Study 1: WD 1 and 30		Study 2: WD1 only
**Binge drinking**			
Average Total Intake	**adolescent > adults** females > males		**adolescents > adults** (males only)
BACs	adolescent > adults females > males		ND
	**Early Withdrawal**	**Late Withdrawal**	**Early Withdrawal**
**Tests for negative affect**			
Latency to enter the light side	EtOH = H2O	EtOH = H2O	EtOH = H2O
Time spent in the light side	EtOH > H2O (adolescents only) **EtOH < H2O** **(adults only)**	EtOH = H2O	**EtOH < H2O** **(adults only)**
Entries into the light side	EtOH = H2O	EtOH = H2O	EtOH = H2O
Latency to bury marbles	ND	ND	EtOH < H2O
Number of marbles buried	EtOH = H2O	EtOH = H2O	EtOH = H2O
Latency to immobility	EtOH > H2O	EtOH = H2O	EtOH = H2O
Time spent immobile	EtOH > H2O (adolescent males only) EtOH < H2O (adult males only)	EtOH = H2O	EtOH = H2O
Immobile Episodes	EtOH > H2O (adult females only) EtOH > H2O (adolescent males only)	EtOH < H2O (adolescents only) EtOH < H2O (males only)	EtOH = H2O
**Morris water maze**			
Latency to platform during the flag test	EtOH = H2O	EtOH = H2O	
Latency to platform during acquisition	EtOH = H2O	EtOH = H2O	
Latency to platform during probe test	EtOH < H2O (adolescents only)	EtOH = H2O	
Entries to platform location during probe test	EtOH = H2O	EtOH < H2O (adolescents only) EtOH > H2O (adults only)	
Time spent in the NE quadrant	EtOH = H2O	EtOH = H2O	
Latency to new platform location	EtOH = H2O	EtOH = H2O	
**Radial arm water maze**			
Reference memory errors	EtOH = H2O	EtOH = H2O	
Working memory correct errors	EtOH = H2O	Days 2 and 3: EtOH < H2O	
Working memory incorrect errors	Days 2, 4 and 5: EtOH > H2O (adolescents only) Day 3: EtOH < H2O (adults only)	Day 2: EtOH < H2O	
Chaining episodes	Days 4 and 5: EtOH > H2O (females only)	EtOH = H2O	
Time to locate all platforms	EtOH = H2O	Days 2 and 3: EtOH < H2O	

EtOH-Water differences in behavior that were consistent across the two studies and/or that align with prior published studies by our group are bolded. ND indicates not determined. The mice in Study 2 were only assayed for negative affect.

However, as observed in our prior large study of sex by age interactions in alcohol withdrawal-induced negative affect (Jimenez Chavez et al., 2020), we detected very few alcohol or -age-related differences in negative affect in either of the studies presented herein (see summary in Table 6). Thus, we twice failed to replicate the robust alcohol by age by withdrawal interactions detected for the majority of our dependent variables in our earlier studies employing a single sex (Lee et al., 2016, 2017b, 2018a,b; Szumlinski et al., 2019). As chemosensory social stimuli from females can affect anxiety-like behavior in males (Aikey et al., 2002; Fernández-Guasti and Martínez-Mota, 2005; Frye et al., 2008), both of the experiments herein tested males and females on different days to mitigate this influence. Thus, gonadal pheromones from mice of the opposite sex during testing cannot readily account for the relatively weak effects of alcohol withdrawal upon our measures of negative affect in the present studies. Likewise, as female mice have historically been housed in the same colony room as male mice, either under ventilated or filter-top-type caging over the years that we have been conducting binge-drinking studies in mice, it is also unlikely that gonadal pheromones from mice of the opposite sex in the colony room can account for the relatively weak effects of alcohol observed in the present studies.

It is interesting to note that we detected more male-selective effects of alcohol withdrawal in the present large-scale study (Table 6), compared to that previous employing concurrent testing of male and female subjects (Jimenez Chavez et al., 2020). As highlighted in Table 6, male-selective alcohol-water differences were noted for the entries into the light-side in the light-dark shuttle box test (WD1), the time spent immobile in the forced swim (WD1), and the number of immobile episodes (both WD1 and WD30), while female-selective alcohol-water differences were noted for the number of immobile episodes (WD1) and the number of marbles buried (WD30) (Table 6). Further, the fact that some sex by age interactions for our measures of negative affect were observed when male and female mice are segregated during testing for negative affect indicates that a segregation strategy may prove more fruitful for detecting such interactions be more optimal for detecting sex-selective effects than concurrent testing of both sexes. Admittedly, the smaller scale replicate study was likely insufficiently powered to detect sex by alcohol interactions as we detected only trends for sex-selective alcohol effects (Figure 9). This being said, Sex by Age interactions were noted for the latency to enter the light-side of the light-dark shuttle box (Figure 9C) and the latency to being burying marbles (Figure 9J), in which males exhibited the age-related difference in behavior. However, the simple fact remains that three of our sex differences studies to date (Jimenez Chavez et al., 2020; present study) have yielded less robust and consistent alcohol effects on anxiety- and depressive-like behaviors than our earlier single-sex studies. While it might be argued that the group-housing procedure employed for water control mice in the present larger scale study and that previous (Jimenez Chavez et al., 2020) may have confounded their results, age by alcohol interactions were apparent in earlier single-sex studies using comparable group-housed water-drinking procedures (Lee et al., 2018a; Szumlinski et al., 2019). Moreover, individually housing both the water- and alcohol-drinking mice in the follow-up study herein did not improve experimental outcomes (see Table 6), despite the study being sufficiently powered to detect alcohol by age interactions (*n* = 12/age/drinking history).

At the time we completed the larger scale study herein, we considered two additional procedural factors that might account for the discrepancy across our sex difference (Jimenez Chavez et al., 2020; present study) versus single-sex studies to date (e.g., Lee et al., 2016, 2017a,b, 2018a,b; Szumlinski et al., 2019): (1) the research personnel conducting the study and (2) the location of the behavioral laboratory. However, as both studies of one or both sexes are labor-intensive, they have always been conducted by teams of researchers such that the mice are handled by multiple, different, researchers throughout drinking and are only tested for negative affect by individuals familiar to the mice, with the goal of minimizing experimenter-induced anxiety-like behavior. We followed a similar “team” approach in the larger scale study herein, while both the drinking and behavioral testing procedures employed in the smaller scale study was conducted by a single researcher. Thus, it would not appear that our “team approach” is a major driver of our failure to detect age by alcohol interactions when both sexes are studied.

A more plausible explanation relates to the locations of the colony rooms in which mice consumed alcohol/water and the procedural space employed for behavioral testing. The mice in all our earlier studies (Lee et al., 2016, 2017a,b, 2018a,b; Szumlinski et al., 2019) were housed and drank alcohol in a small satellite vivarium, with testing conducted in several, small, distinct procedural rooms dedicated to a specific behavioral test that were located outside of the vivarium. While the same behavioral equipment and procedures for assaying negative affect continue to be employed, the three most recent studies from our group examining for age by sex interactions in alcohol withdrawal-induced anxiety (Jimenez Chavez et al., 2020, 2022; present study) were all conducted in the main campus vivarium, in large procedural rooms housing multiple apparati, during which groups of mice undergo different tests concurrently in the same room (i.e., tests for marble-burying conducted on the bench along the right side of the room, with tests for light-dark box conducted on the bench along the left side of the room). To minimize the noise associated with daily vivarium routines, we only tested mice for negative affect on weekends when vivarium staff was minimal and the general vivarium traffic low. However, the modular nature of our current behavioral testing space may not be ideal for testing anxiety- and depressive-like behavior in mice. To probe this possibility, each behavioral assay in the smaller, follow-up, study was conducted in distinct rooms within the main campus vivarium and the mice underwent the behavioral procedures in series. As illustrated in Figure 9, the procedural modifications in the second study were sufficient to unmask age differences and/or age by sex interactions for our light-dark shuttle-box and forced swim measures that were not apparent in the larger scale study (see Figures 3, 5, respectively). However, as highlighted in Table 6, we detected fewer alcohol-related effects in the follow-up study than the larger original study. Unfortunately, as our small satellite vivarium no longer exists, we cannot directly compare outcomes from experiments conducted in the main versus satellite vivaria. Given this, we can conclude that segregating the sexes during behavioral testing and sample size, but not necessarily the involvement of a single versus a team of experimenters, the employ of single versus group-housing of water controls and serial versus concurrent behavioral testing appear to influence the manifestation of negative affect during alcohol withdrawal.

### Robust binge-drinking for 2 weeks elicits a few signs of mild cognitive impairment during alcohol withdrawal

The extant human (e.g., Squeglia et al., 2009, 2011a,b; Novier et al., 2015; Cservenka and Brumback, 2017; Huang et al., 2018; Ledesma et al., 2021) and rodent (Salling et al., 2016; Grifasi et al., 2019; Hoffman et al., 2019; Jimenez Chavez et al., 2022; Van Hees et al., 2022) literature indicates that a history of excessive drinking can accelerate cognitive decline and associated neuropathology, with adolescent female binge-drinking humans exhibiting greater neurocognitive anomalies than their male counterparts (e.g., Squeglia et al., 2009, 2011a,b). Given the robust sex- and age-related differences in alcohol intake and BACs observed in the present study (Figure 1), we predicted that adolescent female mice would exhibit the most robust deficits in cognitive function, potentially exhibiting cognitive anomalies as young adults. However, as summarized in Table 6, only one variable across our Morris water maze procedures exhibited alcohol-dependent effects - the number of entries into the former platform location, a measure of spatial recall. These alcohol effects were observed only in later withdrawal (i.e., approximately 60 days following the last drinking day), were of medium effect size (*d*’s∼0.5) and reflected poorer spatial recall by adolescent-onset binge-drinkers, but better spatial recall by adult-onset binge-drinkers (Figure 6N). No other cognitive measure exhibited an alcohol effect that was selective for adolescent-onset binge-drinkers (Table 6). Thus, while non-dependence drinking can alter the expression of Alzheimer’s Disease-related genes in both adolescent and adult B6 mice (Salling et al., 2016; Hoffman et al., 2019), it may be that a 2-week history of binge-drinking under our 2-h procedures during adolescence is insufficient to accelerate cognitive decline. Alternatively, 3.5 months of age may be too early to detect signs of alcohol-induced cognitive decline in mice with a history of adolescent-onset binge-drinking. Arguing in favor of the former (and against the latter) possibly, Van Hees et al. (2022) recently showed that 10 days of binge-drinking during adolescence under 4-h DID procedures [during which alcohol intakes were approximately double those observed in the present study; see Figure 2C in Van Hees et al. (2022)] is sufficient to induce a deficit in novel object recognition when mice are tested 40 days later. It is also possible that the Morris water maze is less sensitive than other cognitive tasks for the detection of alcohol-induced cognitive decline. Indeed, in our prior study of mature adult and aged mice, we detected very few alcohol-related effects in the Morris water maze, while several measures in the radial arm maze were consistently negatively impacted by an alcohol-drinking history (Jimenez Chavez et al., 2022).

Consistent with this, we detected more alcohol effects in the radial arm maze than in the Morris water maze in the present study (Table 6). However, in contrast to older mice (Jimenez Chavez et al., 2022), the alcohol-water differences observed in adolescent- and adult-onset binge-drinking mice were not systematic across maze acquisition. For some variables, alcohol effects were observed for 1–2 days during early learning, for other variables they appeared during the middle of the first week of training and no obvious pattern of effect is apparent from the results of specific alcohol-water comparisons as presented in Table 6. However, a comparison of the shapes of the time-courses for both working memory correct (Figure 6S) and incorrect errors (Figure 6D’) committed by the binge-drinking mice in later withdrawal argues that a binge-drinking history impairs between-session learning in a manner that is independent of both sex and age of drinking-onset. To the best of our knowledge, this study is the first to examine the effects of a history of binge-drinking during adolescence or younger adulthood on radial arm maze performance. As we know that a month of binge-drinking under our 2-h DID procedures is sufficient to induce sex- and age-selective deficits in radial arm maze performance in older mice (Jimenez Chavez et al., 2022), while binge-drinking large amounts of alcohol (6–8 g/kg/day) over a 10-day period during adolescence is sufficient to induce cognitive deficits in early adulthood (Van Hees et al., 2022), future work seeks to determine the relationship between cumulative alcohol intake and cognitive outcomes, with a focus on how individual differences, such as sex and age of drinking-onset, modify this relationship. As a history of alcohol-drinking during adolescence/early adulthood induces microglial activation (Grifasi et al., 2019), as well as increases the expression of markers of Alzheimer’s disease-related neuropathology (e.g., Salling et al., 2016; Hoffman et al., 2019), future work also seeks to relate alcohol-induced cognitive anomalies, even those mild signs observed herein, to indices of neuropathology.

## Conclusion

Herein we show that a 2-week history of binge-drinking by male and female, adult and adolescent, B6 mice induces relatively few signs of negative affect, some of which were sex-selective. Further, this binge-drinking history is sufficient to induce some signs of mild cognitive impairment in both adolescent- and adult-onset binge-drinkers that persist for greater than 1 month following the cessation of drinking.

## Data availability statement

The raw data supporting the conclusions of this article will be made available by the authors, without undue reservation.

## Ethics statement

The animal study was reviewed and approved by the Institutional Animal Care and Use Committee of the University of California Santa Barbara.

## Author contributions

KS and CJ: conceptualization, supervision, formal analysis, writing—original draft preparation, writing—review and editing, and visualization. KS: project administration. CJ, ED, GS, ER, JT-G, JH, SK, AG, CJED, and MC: investigation and writing—review and editing. KS, CJ, and ED: funding acquisition. All authors contributed to the article and approved the submitted version.
